# Genomic Analysis of Stress Response against Arsenic in *Caenorhabditis elegans*

**DOI:** 10.1371/journal.pone.0066431

**Published:** 2013-07-24

**Authors:** Surasri N. Sahu, Jada Lewis, Isha Patel, Serdar Bozdag, Jeong H. Lee, Robert Sprando, Hediye Nese Cinar

**Affiliations:** 1 Division of Virulence Assessment, Food and Drug Administration, Laurel, Maryland, United States of America; 2 Division of Molecular Biology, Food and Drug Administration, Laurel, Maryland, United States of America; 3 Department of Mathematics, Statistics, and Computer Science, Marquette University, Milwaukee, Wisconsin, United States of America; 4 Oak Ridge Institute for Science and Education, Oak Ridge, Tennessee, United States of America; 5 Kyungpook National University (KNU), Daegu, South Korea; 6 Division of Toxicology, Food and Drug Administration, Laurel, Maryland, United States of America; East Carolina University, United States of America

## Abstract

Arsenic, a known human carcinogen, is widely distributed around the world and found in particularly high concentrations in certain regions including Southwestern US, Eastern Europe, India, China, Taiwan and Mexico. Chronic arsenic poisoning affects millions of people worldwide and is associated with increased risk of many diseases including arthrosclerosis, diabetes and cancer. In this study, we explored genome level global responses to high and low levels of arsenic exposure in *Caenorhabditis elegans* using Affymetrix expression microarrays. This experimental design allows us to do microarray analysis of dose-response relationships of global gene expression patterns. High dose (0.03%) exposure caused stronger global gene expression changes in comparison with low dose (0.003%) exposure, suggesting a positive dose-response correlation. Biological processes such as oxidative stress, and iron metabolism, which were previously reported to be involved in arsenic toxicity studies using cultured cells, experimental animals, and humans, were found to be affected in *C. elegans*. We performed genome-wide gene expression comparisons between our microarray data and publicly available *C. elegans* microarray datasets of cadmium, and sediment exposure samples of German rivers Rhine and Elbe. Bioinformatics analysis of arsenic-responsive regulatory networks were done using FastMEDUSA program. FastMEDUSA analysis identified cancer-related genes, particularly genes associated with leukemia, such as *dnj-11*, which encodes a protein orthologous to the mammalian ZRF1/MIDA1/MPP11/DNAJC2 family of ribosome-associated molecular chaperones. We analyzed the protective functions of several of the identified genes using RNAi. Our study indicates that *C. elegans* could be a substitute model to study the mechanism of metal toxicity using high-throughput expression data and bioinformatics tools such as FastMEDUSA.

## Introduction

Arsenic is a metalloid, which is distributed throughout the Earth crust in diverse complex forms with pyrites. Depending on the physicochemical conditions of the environment, arsenic can readily be dissociated from the complex, enter into ground water [1] and be taken up by microorganisms resulting in high levels of bio-availability [1], [2]. In Asia, including India, Bangladesh, Vietnam, Thailand and China millions of people are exposed to arsenic. Two different oxidative states of arsenic, (III) and (V), are available in organic and inorganic forms that correlate with their cytotoxic potentials. Between these two states, compounds with (+3) oxidation state are more toxic to target cells and tissues due to several mechanisms including high affinity for protein thiols or vicinal sulfhydryl groups [3]–[8].

Chronic and/or acute high dose arsenic exposure can cause wide range of health problems including cancer, severe gastrointestinal toxicity, diabetes, cardiovascular disease and even death [5], [8], [9]. Arsenic is considered as a group1 carcinogen, a categorical classification of an agent/mixture, which is definitely carcinogenic to humans [10]. Since carcinogenic metals, including arsenic, tend to be weak mutagens, and they do not directly interact with DNA, several recent studies have suggested that epigenetic regulation may play a role in metal-induced carcinogenesis [11].

Although the metabolism of inorganic arsenic is quite well known, the precise mechanism of arsenic toxicity is not clearly understood. In mammals, a methylation pathway has been proposed for the metabolic processing of inorganic arsenicals. In this pathway, arsenite (iAs^III^) is sequentially converted to monomethylarsonic acid (MMA^v^) and dimethylarsinic acid (DMA^v^) in both humans and laboratory animals including mice and rats. The intermediate arsenicals, MMAIII and DMAIII, also produced in this pathway, are highly toxic and suspected to be responsible for arsenic toxicity [12]. While some steps in this pathway are strictly chemical reactions, others are enzymatically catalyzed. However, work to date has identified one methyltransferase that is clearly a participant in this pathway. Arsenic (+3 oxidation state) methyltransferase (AS3MT)1 catalyzes conversion of iAs to methylated products. AS3MT homologs have not been identified in *C. elegans* genome [13]. Other aspects of arsenic metabolism in *C. elegans* remain to be seen. Arsenic causes oxidative stress, apoptosis and mutagenesis [14]–[16]. Oxidative stress through generation of reactive oxygen species due to arsenic exposure [17]–[20] have been reported in tumor cell lines [21] as well as in normal human cells [22], [23].

While arsenic is mostly documented as an inducing factor in cancers or several other diseases, there is extensive evidence that one form of arsenic, As_2_O_3_, has a potential antitumor effect *in vitro* and *in vivo* [24]–[26]. United States Food and Drug Administration (US-FDA) approved As_2_O_3_ for the treatment of Acute Promyelocytic Leukemia (APL). It's well established that As_2_O_3_ can completely cure ∼80–90% of newly diagnosed APL patient [24]–[26].

*C. elegans*, a model organism that is less complex than the mammalian system while still sharing high genomic homology, provides an excellent model to elucidate the mechanisms of heavy metal toxicity [27]. This soil nematode has been used in toxicology studies, revealing molecular mechanisms of heavy metal toxicity [28], [29], [30]. Therefore, the *C. elegans* model system is valuable for the investigation of metal toxicity and may be particularly useful for examining gene-environment interactions. Several toxicity endpoints are well documented in the nematode, including growth rate, lifespan, reproduction, and feeding [31], [32]. Acute toxicity can also be assessed in the nematode using altered gene expression levels, as well as behavioral endpoints, such as locomotion, and head thrashing [33]–[37]. Several cellular stress response systems such as the glutathione (GSH), metallothioneins (MTs), heat shock proteins (HSPs), as well as a variety of pumps and transporters are found to work to detoxify and excrete metals in *C. elegans* [27]. Previously, whole genome *C. elegans* DNA microarray and RNAi analysis were used to explore global changes in this nematode to understand mechanisms involved in resistance to cadmium toxicity [38].

In this study we used *C. elegans* whole genome expression microarrays to examine global changes in the nematode transcription profile upon arsenic exposure. Bioinformatics analysis of regulatory networks was done using FastMEDUSA. We analyzed the protective functions of several of the identified genes using RNAi. Molecular players previously associated with arsenic exposure in higher organisms were identified at a global level, confirming the effectiveness of the study. Moreover, we identified evolutionary conserved genes which were not previously associated with arsenic exposure, but associated with carcinogenesis.

## Materials and Methods

### Bacterial strains, media and culture conditions

Eight RNAi bacterial strains were used in this study including: *sdz-8, ftn-1, hsp-70, numr-1, aip-1, gst-37, gcs-1*, and L4440 (empty vector control) [39].

### C. elegans strains and maintenance

Strains N2, NL2099 *rrf-3(pk1426)*, VC1642 *dnj-11(gk1025)*, and VC392 *dac-1(gk211)* were acquired from *Caenorhabditis* Genetics Center (CGC). Strains were maintained at 22°C. The wild type Bristol strain N2, was cultured in *C. elegans* habitation media (CeHM) in tissue culture flasks on a platform shaker [40]. Nematodes were bleached (0.5M NaOH, 1% Hypochlorite) to collect eggs which were incubated in M9 media for 24 hours to bring them to synchronized L1 stage and then transferred to *C. elegans* habitation media (CeHM).

### Arsenic treatment for microarray experiments

Synchronized L1 stage animals were collected by spinning at 800 rpm for five minutes and transferred to Sodium arsenite containing (0.03% and 0.003% w/v) CeHM media and incubated at 22°C for 6 hours.

### RNA Isolation

After arsenic treatment animals were collected and washed in M9 buffer, RNA was extracted using TRIzol reagent (Invitrogen). Residual genomic DNA was removed by DNase treatment (Ambion, Austin, TX). Three independent RNA isolations were performed with each condition for microarray analysis.

### Microarray Analysis

For each experimental condition, RNA was isolated from three biological replicate samples. cRNA was synthesized from 10 µg of total RNA, and samples were hybridized to the *C. elegans* GeneChip (Affymetrix, Santa Clara, CA) by the US Food and Drug Administration/CFSAN/DMB Microarray Facility following the manufacturers instruction. The chip represents 22,500 transcripts of the expressed *C. elegans* genome based on the December 2005 genome sequence. The data were processed using Partek Genomics Suite, version 6.5 (Copyright © 2010 Partek Inc., St. Louis, MO, USA). The robust multichip averaging (RMA) algorithm was used to normalize and summarize the probe data into probeset expression values. The RMA algorithm performs background correction, normalization, and summarization using PM-only probes (Figure S1). A gene usually maps to several probesets. To convert probeset-level expression data to gene-level, we picked the highest-intensity probeset of each gene. We used ANOVA to compute differentially expressed genes between experimental treatment groups and control using the log transformed normalized intensity values generated from application of the RMA algorithm. We used the FDR for multiple comparison correction. Genes were considered differentially expressed if they had a p-value≤0.05 after correction. The microarray data have been deposited in the GEO repository. Accession number is GSE39012.

### Functional Enrichment Analysis

Genes showing a significant change in expression by microarray analysis (*FDR*<0.05) were analyzed using ‘stats’ R package of R software (R Development Core Team [2012]: A language and environment for statistical computing. R foundation for Statistical Computing, Vienna, Austria. ISBN 3-900051-07-0, URL http://www.R-project.org). Genes were compared against a 21,249 *C. elegans* gene database to identify over-represented Gene Ontology terms. Statistical analysis was performed using chi-square test and the Yates' continuity correction. Significant functional terms were defined as *p*<0.05.

### qRT-PCR

cDNA was synthesized from 5 µg of total RNA using random hexamers and SuperScript II reverse transcriptase (Invitrogen). Real time PCR was performed using SYBR Advantage quantitative PCR premix (Clontech) and gene-specific oligonucleotide primers on the LightCycler (Roche). Primers for qRT-PCR are listed on Table S1. Relative fold-changes for transcripts were calculated using the comparative *C_T_* (2^−ΔΔ*CT*^) method [41]. Cycle thresholds of amplification were determined by Light Cycler software (Roche). All samples were run in triplicates and normalized to GAPDH.

### RNA Interference

*E. coli* DH5α bacterial strains expressing double-stranded *C. elegans* RNA [42] were grown in LB broth containing ampicillin (100 µg/ml) at 37°C and plated onto NGM containing 100 µg/ml ampicillin and 3 mm isopropyl 1-thio-β-d-galactopyranoside (IPTG). RNAi-expressing bacteria were allowed to grow overnight at 37°C. Synchronized L1 stage NL2099 (*rrf-3*) strains were used for RNAi experiments for the functional validation of the differentially expressed genes identified through microarray. NL2099 (*rrf-3*) worms were exposed to fresh RNAi expressing bacterial lawn on NGM agar plates for 48 hours, then washed with M9 and plated on sodium arsenite containing NGM plates with *E.coli* OP50 bacterial lawn, and incubated at 22°C (See ‘*C. elegans* survival assays for arsenic exposure following RNAi’ section below). *L4440* RNAi which contains the RNAi plasmid only was included as a control in all experiments.

### *C. elegans* survival assays for arsenic exposure following RNAi

Sodium arsenite containing (0.03%) nematode growth media (NGM), in 6-cm Petri plates, were prepared for survival assays. The plates contained a lawn of OP50 bacteria as a food source. Plates were incubated overnight at room temperature before animals were added. Worms (L1 stage), treated with RNAi bacteria for 48 hours, were transferred to sodium arsenite containing NGM plates with OP50 bacterial lawns and incubated at 22°C. Around 20–30 L4 stage worms were added to each plate. Total 75 to 100 animals were scored for each condition every 24 h for survival and transferred to fresh bacterial lawns every day to avoid overgrowth by progeny. Assay was continued up to ten days. Animal survival was plotted using Kaplan-Meier survival curves and analyzed by log rank test using Graph Pad Prism (Graph Pad Software, Inc., La Jolla, CA). Survival curves resulting in *p* values of <0.05 relative to control were considered significantly different.

### FastMEDUSA analysis

We used FastMEDUSA [43] to elucidate transcription factors (TFs) that putatively regulate the genome-level responses to high and low levels of arsenic exposure in *C. elegans*. FastMEDUSA applies a machine learning algorithm called boosting to train a predictive model from expression and promoter sequences of genes in a number of experimental conditions. FastMEDUSA uses a list of candidate TFs, the promoter sequences of all the genes and a matrix of discrete expression data as input. To discretize gene expression data, we computed fold change of expression signal of a gene in a sample to the gene's median expression across reference samples. A gene in a sample was called upregulated if the fold change ≥1.5 and downregulated if the fold change is ≤−1.5. Genes having inconsistent expression calls across technical replicates were filtered out. We obtained the list of candidate TFs in *C. elegans* from EDGEdb [44], and obtained 1,000 bp promoter sequence of genes from BioMart [45].

FastMEDUSA potentially builds a different model at each run as it contains some stochastic steps. Thus, we ran FastMEDUSA five times using a different random seed value at each run on the Biowulf cluster at the National Institutes of Health. For each FastMEDUSA run, we computed significance score of TFs as following. First, we computed prediction score for the upregulated genes in the experimental condition based on the original FastMEDUSA model. Then, we remove the TF from the FastMEDUSA model and recomputed the prediction score for the same gene set. The difference between the prediction scores give the significance score of the TF (details in [46]). We selected top 20 TFs with highest significance score. Then we selected top ten *consensus* significant TFs that were selected as significant in at least four out of five runs. To find significant TF-gene associations, we computed the significance score for each TF-gene pair. We selected TF-gene associations that had a significance score≥1 for at least four out of five runs and generated a network of these associations by using Cytoscape [47].

## Results and Discussion

### Arsenic exposure induced genome-wide gene expression changes in *C. elegans*

**Figure 1:**
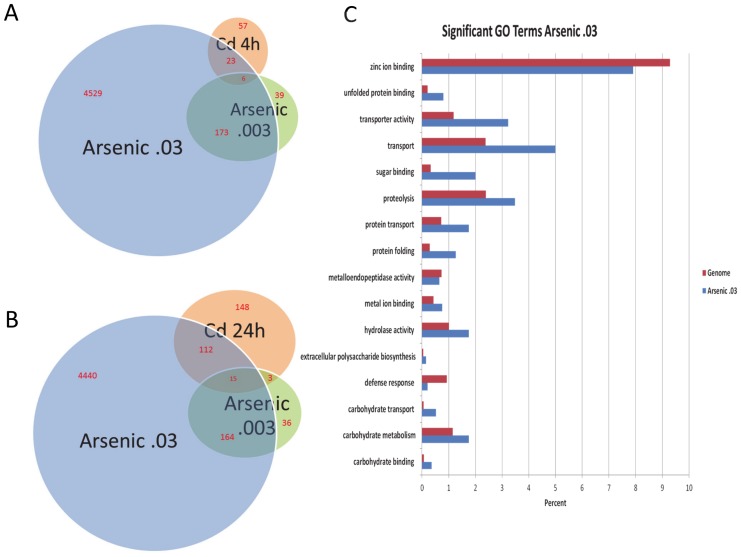
Genome-wide expression profile comparisons of *C. elegans* genes regulated by arsenic and cadmium. (A) A Venn diagram illustrating number of genes expressed at low and high dose arsenic exposure, 4-hour cadmium exposure, and overlap among these data. (B) A Venn diagram illustrating number of genes expressed at low and high dose arsenic exposure, 24-hour cadmium exposure, and overlap among these data. (C) Enrichment of gene ontology categories for genes differentially expressed at high dose arsenic exposure.

Arsenic induced global gene expression has been poorly explored. To study the global gene expression pattern after acute arsenic exposure, we performed a microarray study where wild type L4 stage *C. elegans* (N2) was exposed to sodium arsenite in two different concentrations (0.03% and 0.003% w/v) in CeHM media for 6 hours. Differentially expressed genes were identified (considering fold change (+/−) 1.2 fold, FDR = 0.05 and P<0.05). *C. elegans* gave a strong global gene expression response to sodium arsenite where about one fifth of the genome (4731 genes) was differentially expressed upon high dose (0.03% w/v) exposure. Low dose (0.003% w/v) sodium arsenite led to differential expression in 218 genes, 179 of those were common between the two exposures (Fig. 1). Microarray data were confirmed using qRT-PCR to measure the expression levels of a set of selected genes (Fig. S2).

### Comparison of gene expression changes between high and low levels of sodium arsenite exposure

We exposed worms to two different concentration of sodium arsenite to evaluate the genomic responses to different levels of arsenic exposure. This experimental design allows us to do microarray analysis of dose-response relationships of global gene expression patterns. High dose (0.03%) exposure caused stronger global gene expression changes in comparison with low dose (0.003%) exposure (Fig. 1, Table S2). Two hundred and four genes were up regulated four fold and higher upon high dose exposure, and forty nine genes were up regulated four fold and up upon low dose exposure. Forty six of these were common between these lists (Table 1). Forty three of forty six commonly upregulated genes show dose-response relationship where high levels of sodium arsenite led to higher gene expression levels in *C. elegans* (Table 1). At eight hours exposure we did not observe anatomical level changes in tissue structure, and lethality (data not shown).

**Table 1:** List of genes up-regulated (>4 times) in both high and low dose arsenic exposure.

Gene name	Brief description	Fold-Change (03% vs No Treatment)	*p*-value	Fold-Change (003% vs No treatment)	*p*-value
*numr-1*	unknown	140.416	4.44E-09	25.1465	5.73E-08
*W06H8.2*	unknown	91.5165	2.23E-09	25.2296	1.67E-08
*gst-30*	glutathione S-transferase	64.4519	2.75E-10	12.8297	5.18E-09
*T19D12.3*	unknown	63.3912	2.67E-09	16.5752	2.76E-08
*ftn-1*	ferritin heavy chain	55.3853	2.60E-08	5.7706	3.57E-06
*hsp-70*	heat shock protein 70	53.7595	8.41E-09	14.8787	8.61E-08
*F55G11.2*	unknown	46.5611	1.05E-08	23.5514	3.39E-08
*gst-38*	glutathione S-transferase	44.4153	2.61E-10	25.1488	6.92E-10
*F56D5.3*	NADH oxidase	42.8716	4.55E-07	9.13127	1.04E-05
*clec-3*	unknown	42.2267	1.34E-07	10.7623	1.986E-06
*hsp-16.41*	heat shock protein	37.3042	5.15E-09	21.7315	1.36E-08
*gst-12*	glutathione S-transferase	32.4796	2.51E-08	13.1085	1.52E-07
*hsp-16.2*	heat shock protein	31.7514	6.75E-08	15.6115	2.66E-07
*gst-25*	glutathione S-transferase	29.8987	2.85E-09	6.60598	9.57E-08
*dod-17*	unknown	27.2977	1.12E-08	9.87008	1.01E-07
*Y38E10A.13*	unknown	26.7901	2.41E-06	4.21829	0.0002776
*clec-163*	C-type lectin	26.6706	3.41E-09	6.12515	1.19E-07
*aip-1*	AN-1-like zinc finger-containing protein	20.8251	1.36E-08	4.29368	1.08E-06
*F44E5.4*	Hsp70 family	18.5558	2.86E-09	6.50486	4.08E-08
*clec-2*	C-type lectin	17.5573	1.56E-08	5.14494	4.39E-07
*gst-16*	glutathione S-transferase	16.8344	3.30E-09	8.24937	1.89E-08
*F43E2.5*	methionine sulfoxide-S-reductase (MsrA)	16.6723	5.69E-09	5.49549	1.14E-07
*hsp-16.1*	heat shock protein HSP16-1	14.9708	1.10E-07	6.90193	8.15E-07
*sdz-8*	alcohol dehydrogenase	14.765	1.73E-10	6.55788	1.49E-09
*ZK742.4*	predicted NADH:flavin oxidoreductase	14.2453	9.39E-09	7.42734	5.04E-08
*H20E11.2*	unknown	14.2209	6.13E-09	7.25052	3.53E-08
*gst-5*	glutathione S-transferase	12.8611	3.93E-10	6.47925	2.56E-09
*C32H11.4*	unknown	12.2617	1.28E-11	9.75833	2.26E-11
*Y52E8A.3*	unknown	11.9775	1.94E-09	10.165	2.92E-09
*hsp-16.48*	heat shock protein	11.9585	8.18E-09	7.00906	3.49E-08
*F49F1.6*	containing a signal sequence and ShK toxin domains	10.8365	1.28E-08	4.10812	2.90E-07
*cdr-4*	glutathione S-transferase	10.6628	1.14E-08	4.46537	1.76E-07
*Y40B10A.2*	unknown	9.86761	5.76E-10	4.30897	8.49E-09
*C55A6.6*	alcohol dehydrogenase	9.78048	5.79E-08	5.28538	3.77E-07
*ugt-13*	ugt family	9.63811	1.88E-06	7.71078	3.47E-06
*clec-143*	C-type lectin	9.15882	1.75E-08	4.8121	1.36E-07
*nit-1*	Nitrilase	7.82259	7.22E-08	4.76861	3.72E-07
*gst-4*	glutathione S-transferase	7.60112	9.16E-09	5.85187	2.09E-08
*C17H12.6*	unknown	6.8668	1.65E-07	6.55322	1.91E-07
*clec-9*	C-type lectin	6.4249	1.88E-06	6.405	1.90E-06
*dod-24*	unknown	5.66907	6.82E-09	4.92816	1.13E-08
*gst-7*	glutathione S-transferase	4.92498	7.38E-08	4.22807	1.34E-07
*C32H11.3*	unknown	4.57737	3.54E-07	4.55978	3.60E-07
*C12C8.2*	cystathionine gamma-lyase orthologous to human CTH	4.22098	2.29E-07	4.30506	2.11E-07
*gst-20*	glutathione S-transferase	4.16523	3.23E-07	4.9671	1.61E-07
*F55G1.9*	carboxylate reductase	4.09065	4.49E-09	4.61329	2.75E-09

### Protective function of the subset of the genes up-regulated against arsenic treatment was evaluated using RNAi

**Figure 2:**
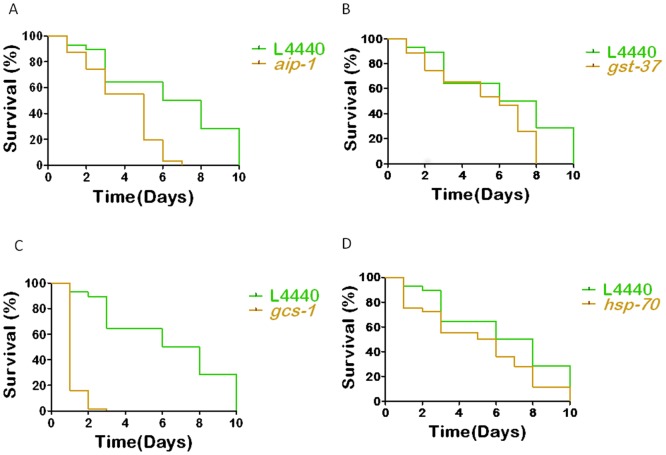
Arsenic induced genes mediate stress response. (A) *aip-1, p<0.0001*, (B) *gst-37, p = 0.0085*, (C) *gcs-1, p<0.0001* and (D) *hsp-70, p = 0.04*, RNAi results in lethality in *C. elegans* exposed to sodium arsenite.

We wanted to test whether knocking down the upregulated genes will affect the sodium arsenite induced lethality in *C. elegans*. Four out of seven genes tested, caused statistically significant increase in lethality upon sodium arsenite exposure when knocked-out via RNAi, suggesting that these genes may have stress response function against arsenic (Fig. 2). Among these genes, *aip-1* encodes an AN-1-like zinc finger-containing protein homologous to arsenite-inducible RNA-associated protein (AIRAP), which is conserved among *C. elegans*, Drosophila, and mammals. AIP-1 is a predicted RNA binding protein that may function in ubiquitin-mediated proteolysis following arsenite treatment. AIP-1 is expressed at high levels in hypodermal and intestinal cells of *C. elegans* following arsenic exposure, and previously shown to protect *C. elegans* and mammalian cells from arsenite toxicity [48]. Our *aip-1* RNAi results agree with the previously published data (Fig. 2A). *gcs-1* encodes the *C. elegans* ortholog of gamma-glutamylecysteine synthetase heavy chain (GCS(h)), which is predicted to function as a phase II detoxification enzyme that catalyzes the rate-limiting first step in glutathione biosynthesis, in a conserved oxidative stress response pathway [49]. Inoue et al [50] showed that the *Caenorhabditis elegans* PMK-1 p38 MAPK pathway regulates the oxidative stress response via the CNC transcription factor SKN-1, leading to phosphorylated SKN-1 accumulation in intestine nuclei, where SKN-1 activates transcription of *gcs-1*. SKN-1 also regulates expression of AIP-1 [51]. We found that most of the *C. elegans* Glutathion S-transferases (GSTs), which are important detoxifying enzymes, responded to arsenic exposure (Table 2). Among these genes, *gst-37*, previously defined as a acrylamide responsive gene in *C. elegans* using expression microarrays [52]. *gst-37* RNAi experiments resulted in increased lethality in arsenic exposure conditions (Figure 2B). In our microarray data several *hsp* (heat-shock protein) genes found to be responsive to sodium arsenite (Table 2). HSP-70 is a member of the hsp70 family of molecular chaperones, involving in general stress response, including response to heat and cadmium exposure, in *C. elegans* [30], [53]. We found that *hsp-70* RNAi leads to increased lethality in arsenic exposure conditions (Figure 2D). Arsenic toxicity leads to induced HSP70 in other systems including *Xenopus laevis* embryos [54], and broiler chickens [55]. Oxidative stress, the central component of heat shock response, is also induced by arsenic [56].

**Table 2:** Oxidative stress and iron metabolism related gene expression is altered upon arsenic exposure in *C. elegans*.

Gene class	Gene symbol	*P*-value	Fold change
Glutathione S-Transferase	*gst-1*	7.05368E-08	3.67142
	*gst-2*	1.23816E-06	16.4022
	*gst-3*	0.000598368	2.02115
	*gst-4*	9.15939E-09	7.60112
	*gst-5*	3.92978E-10	12.8611
	*gst-6*	6.35087E-08	3.62039
	*gst-7*	7.37605E-08	4.92498
	*gst-8*	0.000617048	2.08152
	*gst-9*	1.05337E-06	10.5567
	*gst-10*	1.08689E-05	1.58882
	*gst-12*	2.51366E-08	32.4796
	*gst-13*	2.19643E-08	7.52696
	*gst-14*	3.17455E-06	5.83786
	*gst-16*	3.30181E-09	16.8344
	*gst-19*	4.29809E-08	−8.44166
	*gst-20*	3.22626E-07	4.16523
	*gst-22*	2.83887E-05	1.85819
	*gst-23*	0.00140456	−1.38266
	*gst-25*	2.85006E-09	29.8987
	*gst-26*	1.01402E-08	−7.46037
	*gst-27*	6.8661E-11	−3.00299
	*gst-28*	4.10692E-06	−3.23139
	*gst-29*	0.00104296	−1.92675
	*gst-30*	2.74896E-10	64.4519
	*gst-31*	6.48964E-07	9.26347
	*gst-36*	0.00580448	1.21481
	*gst-38*	2.6131E-10	44.4153
	*gst-39*	1.262E-08	3.78938
	*gst-40*	4.63406E-09	9.47291
	*gst-42*	1.24648E-07	−2.85235
	*gstk-1*	0.000165975	−1.60882
	*gsto-2*	2.68578E-06	4.99268
	*gsto-3*	1.13627E-07	5.62192
Superoxide dismutase	*sod-1*	0.000634797	1.35592
	*sod-2*	0.00234654	−1.27465
	*sod-4*	0.000138537	2.49845
Glutathione diSulfide Reductase	*C46F11.2*	4.28943E-09	3.27018
Flavin-containing MonoOxygenase	*fmo-1*	0.000092521	1.52425
	*fmo-2*	9.40436E-07	3.03595
	*fmo-3*	0.000199546	−1.34887
	*fmo-5*	5.92737E-05	−1.60891
Glutamate Synthase	*W07E11.1*	0.00131395	−1.54529
Catalase	*ctl-1/ctl-3*	1.96717E-06	2.33936
	*ctl-2*	3.94594E-07	−1.81633
Glutathione Peroxidase	*C11E4.1*	9.01179E-05	−2.21564
	*F26E4.12*	0.000013568	2.04773
	*R05H10.5*	9.17508E-06	4.41822
	*R03G5.5*	4.31118E-05	−1.34898
Heat Shock Protein	*hsp-1*	5.78737E-05	1.52653
	*hsp-12.1*	2.25089E-05	−2.3433
	*hsp-12.2*	0.00731473	−1.24178
	*hsp-16.1/hsp-16.11*	1.09759E-07	14.9708
	*hsp-16.2*	6.75136E-08	31.7514
	*hsp-16.41*	5.15451E-09	37.3042
	*hsp-16.48/hsp-16.49*	8.17801E-09	11.9585
	*hsp-17*	1.89686E-05	2.13635
	*hsp-25*	0.0102672	−1.22664
	*hsp-3*	0.00126013	−1.48891
	*hsp-4*	0.000494313	1.39174
	*hsp-43*	8.36411E-06	2.87926
	*hsp-6*	7.8582E-07	4.9375
	*hsp-70*	8.41166E-09	53.7595
	*F44E5.4*	2.85528E-09	18.5558
Hedgehog-like Proteins	*grl-10*	2.49496E-05	−1.94499
	*grl-14*	2.3101E-07	2.2902
	*grl-4*	0.00135253	1.45752
	*grl-7*	2.50635E-05	−1.86634
	*wrt-6*	−1.33577	2.49E-03
	*wrt-1*	−1.38953	1.43E-04
	*grd-12*	−1.41453	1.18E-02
	*wrt-8*	−1.46887	8.54E-03
	*grd-11*	−1.4916	4.22E-03
	*grd-2*	−1.56007	1.09E-03
	*grd-5*	−1.58758	9.18E-06
	*qua-1*	−1.49428	6.25E-04
	*hog-1*	−1.61787	3.75E-05
	*wrt-3*	−1.84712	6.93E-04
	*wrt-4*	−1.95916	6.27E-07
	*wrt-2*	−2.25742	5.30E-05
	*grd-10*	−2.55	3.99E-05
	*grd-3*	−2.9124	1.51E-05
	*grd-1*	−3.13208	4.69E-05
	*wrt-10*	−4.24045	3.56E-08
Transcription Factor	*skn-1*	0.000524389	2.10743
NADH Oxidase	*F56D5.3*	4.55012E-07	42.8716
Mitochondrial Iron Transporter Sideroflexin	*sfxn-2*	4.77799E-05	−2.22696
	*sfxn-2*	0.000478497	−2.32737
	*sfxn-5*	8.57816E-06	−1.976
Ferritin	*ftn-1*	2.59928E-08	55.3853
	*ftn-2*	5.85469E-06	2.36642
Ferroportin	*fpn-1.1*	7.19801E-09	6.49655
	*fpn-1.2*	7.73361E-06	−2.11996

### Oxidative stress- response genes are induced due to sodium arsenite exposure

**Figure 3:**
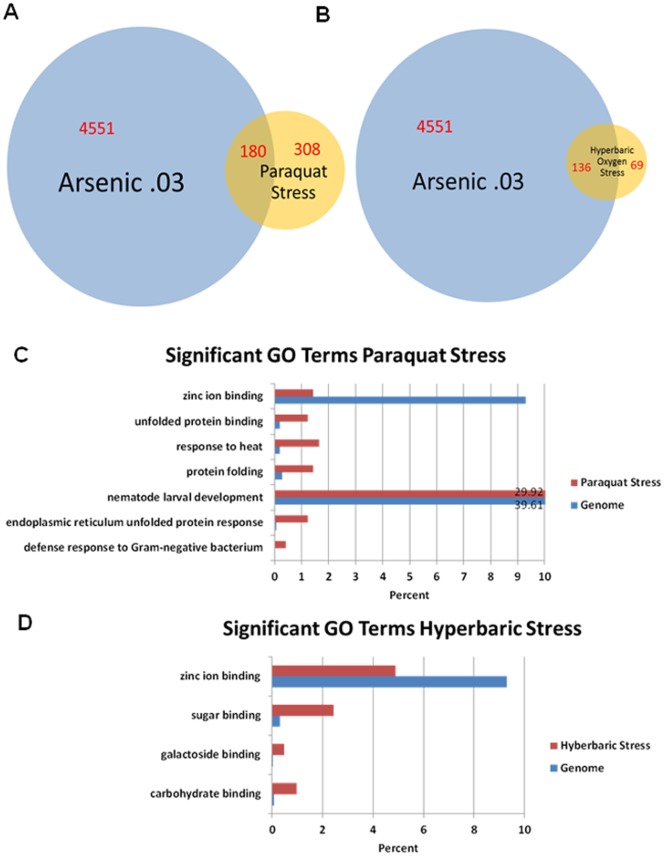
Genome-wide expression profile comparisons of *C. elegans* genes regulated by arsenic and oxidative stress. (A) A Venn diagram illustrating number of genes expressed at high dose arsenic exposure, paraquat exposure, and overlap among these data. (B) A Venn diagram illustrating number of genes expressed at high dose arsenic exposure, hyperbaric oxygen exposure, and overlap among these data. (C) Enrichment of gene ontology categories for genes differentially expressed at paraquat exposure. (D) Enrichment of gene ontology categories for genes differentially expressed at hyperbaric oxygen exposure.

Our microarray data revealed that genomic response of *C. elegans* to sodium arsenite exhibits characteristics of global oxidative stress response (Fig. 3). Oxidative stress from arsenic exposure might result from production of Reactive Oxygen Species (ROS), such as superoxide, hydrogen peroxide, or hydroxyl radical by arsenicals, or from release of iron from ferritin or through induction of heme oxygenase. Increased biosynthesis of defensive enzymes responsive to oxidative stress has been described in both prokaryotes and eukaryotes. We compared our arsenic-response microarray data with previously published *C. elegans* global stress-response results. *C. elegans*' response to both, paraquat- induced stress [57] (Fig. 3A, Table S4), and hyperbaric oxygen-induced stress [58] (Fig. 3B, Table S5) showed significant overlap with our arsenic-response microarray data. We performed Gene Ontology (GO) Term enrichment analysis on our high dose arsenic response data (Figure 1C) along with paraquat and hyperbaric oxygen stress data (Figure 3C and 3D). General stress related GO categories, such as ‘unfolded protein binding’, ‘protein folding’, ‘protein transport’, and proteolysis were found to be enriched under high dose arsenic exposure conditions (Fig. 1C). Some of the protein folding, and transport related GO term enrichments were also present in paraquat stress data but not in hyperbaric oxygen stress data (Fig. 3C, D), suggesting that arsenic and paraquat result in similar functional responses in *C. elegans*. Interestingly, expression of zinc ion binding gene classes was depleted in all of these stresses (Fig. 1C, 3C–D). The essential trace element zinc is broadly required in cellular functions, and disturbances in zinc homeostasis cause a range of health problems that include growth retardation, immunodeficiency, neuronal and sensory dysfunctions [59].

Glutathion S-transferases (GSTs) are essential detoxifying enzymes that constitute up to 10% of cytosolic protein in some mammalian organs, and catalyze the conjugation of reduced glutathione on a wide variety of substrates [60], [61]. This activity detoxifies endogenous compounds such as peroxidised lipids [62]. GSTs may also bind toxins and function as transport proteins [63]. *C. elegans* genome possesses a large number of GST genes. We found that sixty seven percent of (thirty three of forty nine) the *C. elegans gst* genes are differentially expressed upon arsenic exposure (Table 2). Other genes encoding antioxidant enzymes such as catalase, superoxide dismutase, and glutathione peroxidase are differentially expressed in arsenic-exposed *C. elegans* (Table 2). Lynn et. al. [64] reported that arsenite activates NADH oxidase to produce superoxide, which then causes oxidative DNA damage. We found that putative NADH oxidase encoding gene F56D5.3 is upregulated 43 fold in arsenic exposed *C. elegans* (Table 2). Recent studies revealed an association between sonic hedgehog signaling and oxidative stress in several different tissues including rat brain and mouse bone marrow [65], [66]. Twenty of the fifty eight hedgehog related genes of *C. elegans*, found to be differentially expressed upon arsenic exposure (Table 2). Functions of sonic hedgehog signaling genes in arsenic toxcicity and protection remain to be seen.

### Arsenic-induced perturbations in iron metabolism may lead to oxidative stress

Almost all cells utilize iron as a cofactor for essential biochemical activities, such as oxygen transport, energy metabolism and DNA synthesis. However, iron catalyses the propagation of ROS and generation of highly reactive radicals through fenton chemistry, hence, free iron is potentially toxic to cells [67], [68]. Much of the excess intracellular iron is stored in the cytosol, bound to ferritin. Very little is known about the interaction of the species of arsenic with free iron at the cellular level. Release of iron from ferritin is an under investigated possible mechanism of arsenic induced oxidative stress. It has been shown that arsenic species can cause release of iron from horse spleen ferritin *in vitro* [69]. Iron administration into HeLa cells leads to increased ferritin mRNA levels [70]. We found that ferritin encoding genes of *C. elegans*, *ftn-1*, and *ftn-2* are upregulated upon sodium arsenite exposure (table 2). There is strong experimental support suggesting a protective role for ferritin against oxidative stress. Both transcriptional and posttranscriptional mechanisms have been implicated in ferritin induction by oxidants, such as ROS, and nitric oxide [71], [72]. *C. elegans* homologs of iron transporter ferroportin, *fpn-1.1* and *fpn-1.2* are also differentially expressed against sodium arsenite (Table 2). Sideroflexins are recently discovered mitochondrial multiple transmembrane proteins with unknown function, which are associated with iron accumulation in mitochondria [73]. We found that *C. elegans* sideroflexin genes *sfxn-2* and *sfxn-5* are downregulated upon arsenic exposure. Altogether, our data suggest that arsenic may induce perturbations in proteins involved in iron metabolism.

### Genomic response to arsenic versus cadmium in *C. elegans*

Heavy metals such as copper, zinc, cadmium, and metalloids such as arsenic, are major environmental toxicants that are associated with a variety of human diseases. In spite of extensive research on the pathogenesis of human diseases which are linked to environmental heavy metal and metalloid exposure, the fraction of the molecular mechanisms of pathogenesis induced by these agents, that shared, is not known at the genomic level. We compared *C. elegans*' response to cadmium and arsenic using previously published microarray dataset [38]. Using the same threshold for both cadmium and arsenic response datasets (1.5 fold p<0.0001), we found a significant overlap between affected genes (Fig. 1A,B, Fig. S3, Table S3). We performed Gene Ontology (GO) term enrichment analysis on cadmium microarray expression data. Some of the protein folding, and transport related GO term enrichments were present in cadmium response data (Fig. S3A, B). Expression of zinc ion binding gene classes was depleted in cadmium data, similar to our findings regarding arsenic, paraquat stress, and hyperbaric oxygen stress data. GO class of ‘nematode larval development’ was found to be enriched in cadmium response data but not in arsenic response data, suggesting that different developmental consequences may arise against arsenic and cadmium in *C. elegans*.

Robinson et al. reported arsenic- and cadmium-induced toxicogenomic response in mouse embryos undergoing neurulation. They examined the dose-dependent effects of arsenics and cadmium on gene expression in association with increased embryotoxicity in C57BL/6J mouse embryos, and identified overlapping and non-overlapping metal-induced gene expression alterations [74]. They found that 1960 and 775 genes identified to be significantly altered by arsenic and cadmium, respectively (F-test, pb0.0001), and 116 of these genes overlapping between these two populations. Understanding genomic level responses to different heavy metals will help to resolve shared mechanisms of heavy metal- induced diseases.

### Genomic responses of *C. elegans* to environmental contamination can be used as an ecotoxicogenomics tool

**Figure 4:**
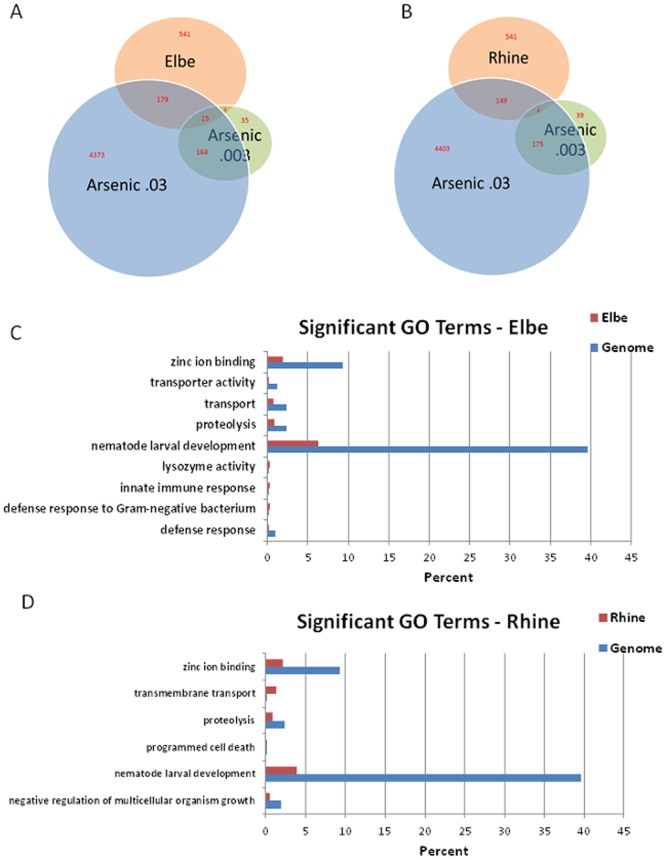
Genome-wide expression profile comparisons of *C. elegans* genes regulated by arsenic and river sediment toxicants. (A) A Venn diagram illustrating number of genes expressed at low and high dose arsenic exposure, Elbe River sediment exposure, and overlap among these data. (B) A Venn diagram illustrating number of genes expressed at low and high dose arsenic exposure, Rhine River sediment exposure, and overlap among these data. (C) Enrichment of gene ontology categories for genes differentially expressed at Elbe River sediment exposure. (D) Enrichment of gene ontology categories for genes differentially expressed at Rhine River sediment exposure.

Arsenic is ubiquitous throughout the earth crust in different complex forms with pyrites [75], can easily dissociate from the complex and enter into ground water [1], and be taken up by microorganisms resulting in high levels of bio-availability [1], [2]. Because of these properties, arsenic is considered as an important environmental toxin. Menzel et al. used *C. elegans* as a bio-monitor to characterize sediment toxicity of German rivers Rhine and Elbe [76]. In that study, *C. elegans* were exposed to sediments of three German rivers, Danube, Rhine, Elbe; Danube being the cleanest, and Elbe is the most contaminated among the three rivers, based on chemical properties of the sediments, including arsenic levels. Using expression profile of *C. elegans* exposed to Danube sediment as a reference, they identified that 748 and 694 transcripts were significantly altered in Elbe and Rhine exposed animals, respectively. We wanted to address the question of how an expression profile identified against a particular pollutant, in our case arsenic, would correlate with an expression profile identified against contaminated river sediments. We found bigger overlap between global arsenic response and response to Elbe, which is the most contaminated river in this study, with higher arsenic levels (Fig. 4, Table S6, S7). These results indicate that *C. elegans* may be used as an environmental bio-monitor, and meta- analysis of publicly available *C. elegans* expression microarray data will provide a platform to gain insights into complex environmental issues.

### Discovering transcription factors of genomic response to arsenic exposure in *C. elegans* using FastMEDUSA

We utilized FastMEDUSA to compute TFs involved in the transcriptional response against high (0.03%) and low (0.003%) concentrations of arsenic in *C. elegans* (see Materials and Methods). We predicted ten consensus-significant TFs associated with high concentration of arsenic (Table 3). As the transcriptional response of *C. elegans* to low concentrations of arsenic was very minimal, FastMEDUSA did not find any significant TFs associated with this condition. We also predicted significant TF-gene associations based on FastMEDUSA models and plotted them in a network (Figure S4, Table S8).

**Table 3:** Transcription factors that are predicted to be involving in the response to arsenic exposure by FastMEDUSA.

Gene name	Description	Anatomic expression pattern
*dac-1*	Ortholog of SKI/SNO/DAC family of proteins	nervous system, hypodermal seam cells
*nhr-35*	Nuclear hormone receptor	Intestine
*C32D5.1*	Putative DNA binding domain	Unknown
*nhr-45*	Nuclear hormone receptor	Unknown
*dnj-11*	Ortholog of the mammalian ZRF1/MIDA1/MPP11/DNAJC2 family	pharynx, intestine, muscle, nervous system, reproductive system
*mxl-2*	Max-Like protein X - bHLH-Zip family protein	hypodermis, intestine
*R06C1.6*	Uncharacterized protein	Unknown
*nhr-61*	Nuclear hormone receptor	Unknown
*zip-4*	Putative C/EBP protein, divergent ortologue to human CEBPA-Mutated in acute myeloid leukemia	Weak general expression and higher expression in pharynx and somatic gonad
*ztf-22*	Zinc finger putative Transcription Factor family	Nervous system, head muscles, intestine

**Figure 5:**
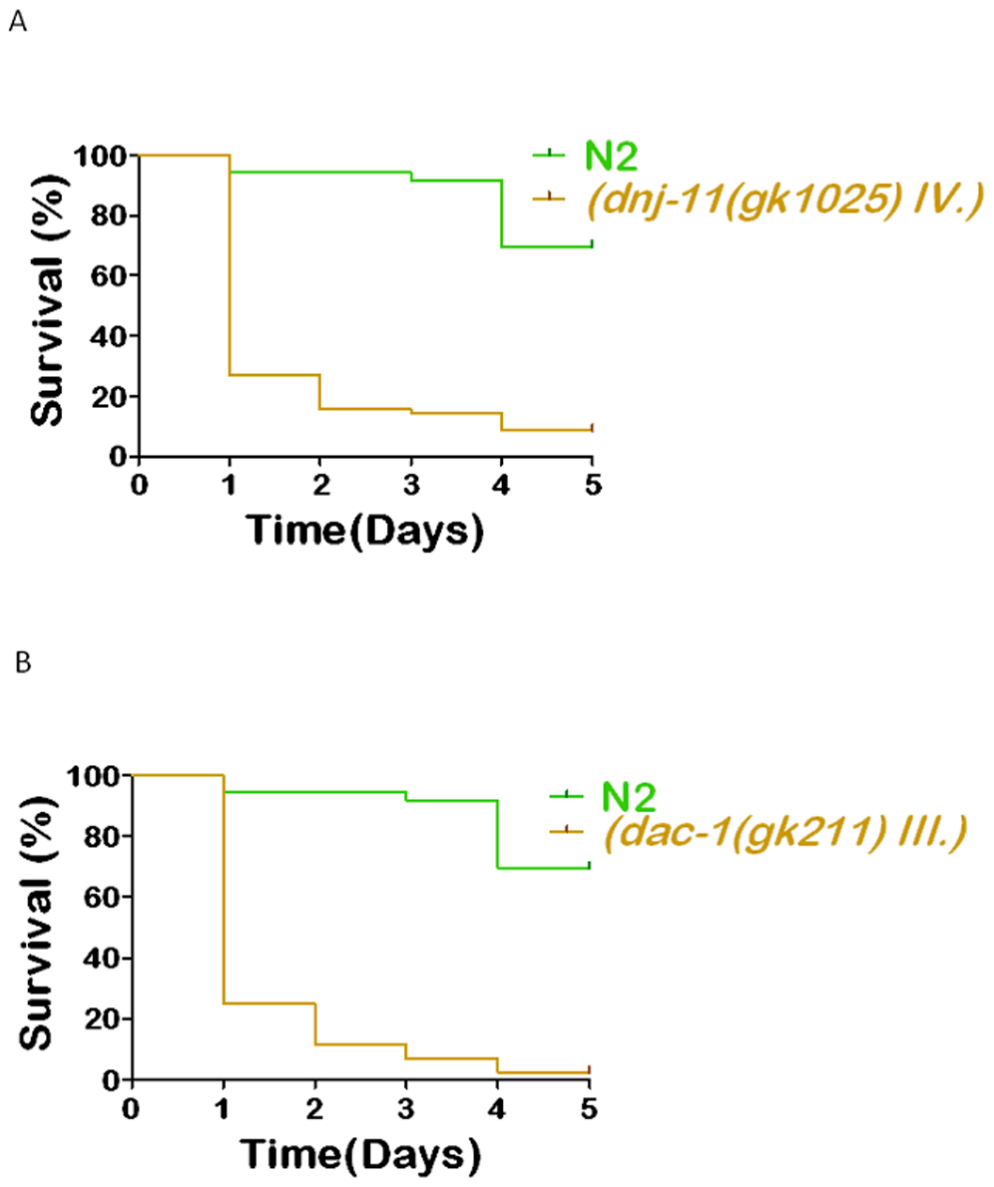
Lethality assays of knock-downs of the FastMedusa identified arsenic responsive regulatory genes. (Assays are performed on arsenic exposure conditions.) (A) *dnj-11(gk1025)*, *p*<0.0001, (B) and *dac-1(gk211)*, *p*<0.0001.

Two of the predicted significant TFs, *dnj-11*and *dac-1*, were tested for their contribution to arsenic stress response. We found that loss of function of these genes using RNAi exhibited increased lethality suggesting that these genes induce the stress response in *C. elegans* (Fig. 5). *dnj-11* encodes a protein containing DnaJ and Myb domains that is orthologous to the mammalian ZRF1/MIDA1/MPP11/DNAJC2 family of ribosome-associated molecular chaperones (Wormbase). MPP11 was identified as a leukemia-associated antigen, and expression of this gene is up-regulated during leukemic blasts in patients [77]. In rats, MPP11 homolog MIDA1 was identified to induce humoral immune responses in glioma, and immunization with MIDA1 containing plasmid resulted in a significant suppression of tumor growth in immunized animals [78], [79].

Chromosomal defects involving MPP11 are associated with primary head and neck squamous cell tumors [80]. Interestingly, a recent study revealed that As_2_O_3_ had anti-cancer effects on both cultured oral squamous cell carcinoma (OSCC) cells and OSCC xenografts by inhibiting cell growth, suppressing angiogenesis and inducing apoptosis [81]. There is extensive evidence that arsenic trioxide (As_2_O_3_), has a potential role of antitumor effect *in vitro* and *in vivo* [24]–[26]. US Food and Drug Administration approved As_2_O_3_ for the treatment of Acute Promyelocytic Leukemia (APL). It's well established that As_2_O_3_ can cure ∼80–90% of newly diagnosed APL patients [24]–[26]. Precise molecular mechanism of the therapeutic effect of As_2_O_3_ is not known. Our results suggest a molecular mechanism for the therapeutic effect of As_2_O_3_, such that, As_2_O_3_ may regulate MPP11 expression, which may stimulate immune responses lead to killing of leukemic blast cells, and squamous cell carcinoma cells.

*dac-1* encodes the *C. elegans* ortholog of Dachshund, a transcriptional regulator of the SKI/SNO/DAC family of proteins first described in Drosophila. The altered expression of DACH1, a Drosophila Dachshund homolog, has been associated with tumor progression and metastasis in human breast, prostate, ovarian and endometrial cancers [82]–[86]. Another arsenic response gene identified via FastMEDUSA is *zip-4*, a putative C/EBP protein, divergent orthologue to human CEBPA gene, which is mutated in acute myeloid leukemia [87]. We also identified several *C. elegans* nuclear receptors (NRs) as arsenic responsive genes using Fast MEDUSA. Nuclear receptors (NRs) encompass a family of transcription factors often regulated by small lipophilic molecules, such as steroids, retinoids, bile and fatty acids, that mediate endocrine control [88]. *C. elegans* has a large family of NRs, containing 284 of these receptors in its genome (wormbook). A large percentage of human cancers, particularly breast, prostate, and endometrial cancers, rely on steroid production for initial growth [89], [90]. Our data suggest that induction of NRs via arsenic may contribute increased incidence of cancers in arsenic exposed human populations.

## Supporting Information

Figure S1 **Boxplots which depict A) row data, and B) data after normalization. PM only probe set signal was applied.**
(TIF)

Figure S2 **qRT-PCR results for selected high ranker genes.**
(TIF)

Figure S3 **GO term enrichments with cadmium in** ***C. elegans*** **.** (A) Enrichment of GO categories for 4-hour cadmium exposure. (B) Enrichment of GO categories for 24-hour cadmium exposure.
(TIF)

Figure S4 **Significant transcription factor- gene interactions in high arsenic condition.** The color of the nodes represent the overall expression of the gene (green: down-regulated, red: up-regulated). The size of vertices is proportional to their degree (i.e., number of edges incident on them). Each node is labeled with the corresponding gene or TF's name. Rounded squares represent transcription factors, and circles represent putative target genes of these transcription factors. The layout of the network was generated manually on Cytoscape.
(TIF)

Table S1 **List of primers used for qRT-PCR.**
(DOCX)

Table S2 **List of genes differentially expressed in both high and low dose arsenic exposure (+/−1.5 fold).**
(DOCX)

Table S3 **List of genes differentially expressed in both, high dose arsenic, and cadmium 24-hour exposures (+/−1.5 fold).**
(DOCX)

Table S4 **List of genes differentially expressed in both, high dose arsenic, and paraquat exposures (+/−1.5 fold).**
(DOCX)

Table S5 **List of genes differentially expressed in both, high dose arsenic, and hyperbaric oxygen exposures (+/−1.5 fold).**
(DOCX)

Table S6 **List of genes differentially expressed in both, high dose arsenic exposure and Elbe River sediment exposures (+/−1.5 fold).**
(DOCX)

Table S7 **List of genes differentially expressed in both, high dose arsenic exposure and Rhine River sediment exposures (+/−1.5 fold).**
(DOCX)

Table S8 **Putative targets of top ten transcription factors that are predicted to be involving in the response to arsenic exposure by FastMEDUSA.**
(DOCX)

## References

[1] Oremland RS, Kulp TR, Blum JS, Hoeft SE, Baesman S, et al (2005) A microbial arsenic cycle in a salt-saturated, extreme environment. Science 308: 1305–1308 308/5726/1305 [pii];10.1126/science.1110832 [doi]. 15919992 10.1126/science.1110832

[2] Bryan CG, Marchal M, Battaglia-Brunet F, Kugler V, Lemaitre-Guillier C, et al (2009) Carbon and arsenic metabolism in Thiomonas strains: differences revealed diverse adaptation processes. BMC Microbiol 9: 127 1471-2180-9-127 [pii];10.1186/1471-2180-9-127 [doi]. 19549320 10.1186/1471-2180-9-127 PMC2720973

[3] Kaltreider RC, Davis AM, Lariviere JP, Hamilton JW (2001) Arsenic alters the function of the glucocorticoid receptor as a transcription factor. Environ Health Perspect 109: 245–251 sc271_5_1835 [pii]. 10.1289/ehp.01109245 PMC1240242 11333185

[4] Liu SX, Athar M, Lippai I, Waldren C, Hei TK (2001) Induction of oxyradicals by arsenic: implication for mechanism of genotoxicity. Proc Natl Acad Sci U S A 98: 1643–1648 10.1073/pnas.031482998 [doi];031482998 [pii]. 11172004 10.1073/pnas.031482998 PMC29310

[5] Dilda PJ, Hogg PJ (2007) Arsenical-based cancer drugs. Cancer Treat Rev 33: 542–564 S0305-7372(07)00073-4 [pii];10.1016/j.ctrv.2007.05.001 [doi]. 17624680 10.1016/j.ctrv.2007.05.001

[6] Ghosh P, Banerjee M, Giri AK, Ray K (2008) Toxicogenomics of arsenic: classical ideas and recent advances. Mutat Res 659: 293–301 S1383-5742(08)00082-3 [pii];10.1016/j.mrrev.2008.06.003 [doi]. 18638567 10.1016/j.mrrev.2008.06.003

[7] Platanias LC (2009) Biological responses to arsenic compounds. J Biol Chem 284: 18583–18587 R900003200 [pii];10.1074/jbc.R900003200 [doi]. 19363033 10.1074/jbc.R900003200 PMC2707240

[8] Ralph SJ (2008) Arsenic-based antineoplastic drugs and their mechanisms of action. Met Based Drugs 2008: 260146 10.1155/2008/260146 [doi]. 18431449 10.1155/2008/260146 PMC2292810

[9] Tapio S, Grosche B (2006) Arsenic in the aetiology of cancer. Mutat Res 612: 215–246 S1383-5742(06)00020-2 [pii];10.1016/j.mrrev.2006.02.001 [doi]. 16574468 10.1016/j.mrrev.2006.02.001

[10] World Health Organization International Agency for Research on Cancer [IARC] (2004) Some drinking-water disinfectants and contaminants, including arsenic. IARC Monogr Eval Carcinog Risks Hum 84: 1–477. 15645577 PMC7682301

[11] Chervona Y, Arita A, Costa M (2012) Carcinogenic metals and the epigenome: understanding the effect of nickel, arsenic, and chromium. Metallomics 4: 619–627 10.1039/c2mt20033c. 22473328 10.1039/c2mt20033c PMC3687545

[12] Watanabe T, Hirano S (2012) Metabolism of arsenic and its toxicological relevance. Arch Toxicol 10.1007/s00204-012-0904-5. 10.1007/s00204-012-0904-5 22811022

[13] Thomas DJ, Li J, Waters SB, Xing W, Adair BM, et al (2007) Arsenic (+3 oxidation state) methyltransferase and the methylation of arsenicals. Exp Biol Med (Maywood) 232: 3–13 232/1/3. 17202581 PMC2408740

[14] Banerjee N, Banerjee M, Ganguly S, Bandyopadhyay S, Das JK, et al (2008) Arsenic-induced mitochondrial instability leading to programmed cell death in the exposed individuals. Toxicology 246: 101–111 S0300-483X(08)00002-4 [pii];10.1016/j.tox.2007.12.029 [doi]. 18304716 10.1016/j.tox.2007.12.029

[15] Buttke TM, Sandstrom PA (1994) Oxidative stress as a mediator of apoptosis. Immunol Today 15: 7–10. 8136014 10.1016/0167-5699(94)90018-3

[16] Kumagai Y, Sumi D (2007) Arsenic: signal transduction, transcription factor, and biotransformation involved in cellular response and toxicity. Annu Rev Pharmacol Toxicol 47: 243–262 10.1146/annurev.pharmtox.47.120505.105144 [doi]. 17002598 10.1146/annurev.pharmtox.47.120505.105144

[17] Flora SJ (1999) Arsenic-induced oxidative stress and its reversibility following combined administration of N-acetylcysteine and meso 2,3-dimercaptosuccinic acid in rats. Clin Exp Pharmacol Physiol 26: 865–869. 10561806 10.1046/j.1440-1681.1999.03157.x

[18] Pi J, Yamauchi H, Kumagai Y, Sun G, Yoshida T, et al (2002) Evidence for induction of oxidative stress caused by chronic exposure of Chinese residents to arsenic contained in drinking water. Environ Health Perspect 110: 331–336 sc271_5_1835 [pii]. 11940449 10.1289/ehp.02110331 PMC1240794

[19] Pi J, Horiguchi S, Sun Y, Nikaido M, Shimojo N, et al (2003) A potential mechanism for the impairment of nitric oxide formation caused by prolonged oral exposure to arsenate in rabbits. Free Radic Biol Med 35: 102–113 S0891584903002697 [pii]. 12826260 10.1016/s0891-5849(03)00269-7

[20] Wu MM, Chiou HY, Wang TW, Hsueh YM, Wang IH, et al (2001) Association of blood arsenic levels with increased reactive oxidants and decreased antioxidant capacity in a human population of northeastern Taiwan. Environ Health Perspect 109: 1011–1017 sc271_5_1835 [pii]. 11675266 10.1289/ehp.011091011 PMC1242077

[21] Woo SH, Park IC, Park MJ, Lee HC, Lee SJ, et al (2002) Arsenic trioxide induces apoptosis through a reactive oxygen species-dependent pathway and loss of mitochondrial membrane potential in HeLa cells. Int J Oncol 21: 57–63. 12063550

[22] Barchowsky A, Dudek EJ, Treadwell MD, Wetterhahn KE (1996) Arsenic induces oxidant stress and NF-kappa B activation in cultured aortic endothelial cells. Free Radic Biol Med 21: 783–790 0891584996001748 [pii]. 8902524 10.1016/0891-5849(96)00174-8

[23] Barchowsky A, Klei LR, Dudek EJ, Swartz HM, James PE (1999) Stimulation of reactive oxygen, but not reactive nitrogen species, in vascular endothelial cells exposed to low levels of arsenite. Free Radic Biol Med 27: 1405–1412 S0891-5849(99)00186-0 [pii]. 10641735 10.1016/s0891-5849(99)00186-0

[24] Douer D, Tallman MS (2005) Arsenic trioxide: new clinical experience with an old medication in hematologic malignancies. J Clin Oncol 23: 2396–2410 23/10/2396 [pii];10.1200/JCO.2005.10.217 [doi]. 15800332 10.1200/JCO.2005.10.217

[25] Sanz MA, Grimwade D, Tallman MS, Lowenberg B, Fenaux P, et al (2009) Management of acute promyelocytic leukemia: recommendations from an expert panel on behalf of the European LeukemiaNet. Blood 113: 1875–1891 blood-2008-04-150250 [pii];10.1182/blood-2008-04-150250 [doi]. 18812465 10.1182/blood-2008-04-150250

[26] Tallman MS, Nabhan C, Feusner JH, Rowe JM (2002) Acute promyelocytic leukemia: evolving therapeutic strategies. Blood 99: 759–767. 11806975 10.1182/blood.v99.3.759

[27] Martinez-Finley EJ, Aschner M (2011) Revelations from the Nematode Caenorhabditis elegans on the Complex Interplay of Metal Toxicological Mechanisms. J Toxicol 2011: 895236 10.1155/2011/895236. 21876692 10.1155/2011/895236 PMC3157827

[28] Slice LW, Freedman JH, Rubin CS (1990) Purification, characterization, and cDNA cloning of a novel metallothionein-like, cadmium-binding protein from Caenorhabditis elegans. J Biol Chem 265: 256–263. 2294106

[29] Freedman JH, Slice LW, Dixon D, Fire A, Rubin CS (1993) The novel metallothionein genes of Caenorhabditis elegans. Structural organization and inducible, cell-specific expression. J Biol Chem 268: 2554–2564. 8428932

[30] Liao VH, Freedman JH (1998) Cadmium-regulated genes from the nematode Caenorhabditis elegans. Identification and cloning of new cadmium-responsive genes by differential display. J Biol Chem 273: 31962–31970. 9822667 10.1074/jbc.273.48.31962

[31] Jones D, Candido EP (1999) Feeding is inhibited by sublethal concentrations of toxicants and by heat stress in the nematode Caenorhabditis elegans: relationship to the cellular stress response. J Exp Zool 284: 147–157 10.1002/(SICI)1097-010X(19990701)284:2<147::AID-JEZ4>3.0.CO;2-Z [pii]. 10404644 10.1002/(sici)1097-010x(19990701)284:2<147::aid-jez4>3.3.co;2-q

[32] Boyd WA, Cole RD, Anderson GL, Williams PL (2003) The effects of metals and food availability on the behavior of Caenorhabditis elegans. Environ Toxicol Chem 22: 3049–3055. 14713049 10.1897/02-565

[33] Anbalagan C, Lafayette I, Antoniou-Kourounioti M, Haque M, King J, et al (2012) Transgenic nematodes as biosensors for metal stress in soil pore water samples. Ecotoxicology 21: 439–455 10.1007/s10646-011-0804-0 [doi]. 22037694 10.1007/s10646-011-0804-0 PMC3277692

[34] Johnson TE, Nelson GA (1991) Caenorhabditis elegans: a model system for space biology studies. Exp Gerontol 26: 299–309. 1915699 10.1016/0531-5565(91)90024-g

[35] Dhawan R, Dusenbery DB, Williams PL (1999) Comparison of lethality, reproduction, and behavior as toxicological endpoints in the nematode Caenorhabditis elegans. J Toxicol Environ Health A 58: 451–462. 10616193 10.1080/009841099157179

[36] Anderson GL, Cole RD, Williams PL (2004) Assessing behavioral toxicity with Caenorhabditis elegans. Environ Toxicol Chem 23: 1235–1240. 15180374 10.1897/03-264

[37] Anderson GL, Boyd WA, Williams PL (2001) Assessment of sublethal endpoints for toxicity testing with the nematode Caenorhabditis elegans. Environ Toxicol Chem 20: 833–838. 11345460

[38] Cui Y, McBride SJ, Boyd WA, Alper S, Freedman JH (2007) Toxicogenomic analysis of Caenorhabditis elegans reveals novel genes and pathways involved in the resistance to cadmium toxicity. Genome Biol 8: R122 gb-2007-8-6-r122 [pii];10.1186/gb-2007-8-6-r122 [doi]. 17592649 10.1186/gb-2007-8-6-r122 PMC2394766

[39] Fraser AG, Kamath RS, Zipperlen P, Martinez-Campos M, Sohrmann M, et al (2000) Functional genomic analysis of C. elegans chromosome I by systematic RNA interference. Nature 408: 325–330 10.1038/35042517 [doi]. 11099033 10.1038/35042517

[40] Sprando RL, Olejnik N, Cinar HN, Ferguson M (2009) A method to rank order water soluble compounds according to their toxicity using Caenorhabditis elegans, a Complex Object Parametric Analyzer and Sorter, and axenic liquid media. Food Chem Toxicol 47: 722–728 S0278-6915(09)00008-8 [pii];10.1016/j.fct.2009.01.007 [doi]. 19162123 10.1016/j.fct.2009.01.007

[41] Schmittgen TD, Livak KJ (2008) Analyzing real-time PCR data by the comparative C(T) method. Nat Protoc 3: 1101–1108. 18546601 10.1038/nprot.2008.73

[42] Kamath RS, Fraser AG, Dong Y, Poulin G, Durbin R, et al (2003) Systematic functional analysis of the Caenorhabditis elegans genome using RNAi. Nature 421: 231–237 10.1038/nature01278 [doi];nature01278 [pii]. 12529635 10.1038/nature01278

[43] Bozdag S, Li A, Wuchty S, Fine HA (2010) FastMEDUSA: a parallelized tool to infer gene regulatory networks. Bioinformatics 26: 1792–1793 btq275 [pii];10.1093/bioinformatics/btq275 [doi]. 20513661 10.1093/bioinformatics/btq275 PMC2894517

[44] Barrasa MI, Vaglio P, Cavasino F, Jacotot L, Walhout AJ (2007) EDGEdb: a transcription factor-DNA interaction database for the analysis of C. elegans differential gene expression. BMC Genomics 8: 21 1471-2164-8-21 [pii];10.1186/1471-2164-8-21 [doi]. 17233892 10.1186/1471-2164-8-21 PMC1790901

[45] Haider S, Ballester B, Smedley D, Zhang J, Rice P, et al (2009) BioMart Central Portal–unified access to biological data. Nucleic Acids Res 37: W23–W27 gkp265 [pii];10.1093/nar/gkp265 [doi]. 19420058 10.1093/nar/gkp265 PMC2703988

[46] Kundaje A, Xin X, Lan C, Lianoglou S, Zhou M, et al (2008) A predictive model of the oxygen and heme regulatory network in yeast. PLoS Comput Biol 4: e1000224 10.1371/journal.pcbi.1000224 [doi]. 19008939 10.1371/journal.pcbi.1000224 PMC2573020

[47] Shannon P, Markiel A, Ozier O, Baliga NS, Wang JT, et al (2003) Cytoscape: a software environment for integrated models of biomolecular interaction networks. Genome Res 13: 2498–2504 10.1101/gr.1239303;13/11/2498. 14597658 10.1101/gr.1239303 PMC403769

[48] Sok J, Calfon M, Lu J, Lichtlen P, Clark SG, et al (2001) Arsenite-inducible RNA-associated protein (AIRAP) protects cells from arsenite toxicity. Cell Stress Chaperones 6: 6–15. 11525245 10.1379/1466-1268(2001)006<0006:airapa>2.0.co;2 PMC434377

[49] Liao VH, Yu CW (2005) Caenorhabditis elegans gcs-1 confers resistance to arsenic-induced oxidative stress. Biometals 18: 519–528 10.1007/s10534-005-2996-3 [doi]. 16333752 10.1007/s10534-005-2996-3

[50] Inoue H, Hisamoto N, An JH, Oliveira RP, Nishida E, et al (2005) The C. elegans p38 MAPK pathway regulates nuclear localization of the transcription factor SKN-1 in oxidative stress response. Genes Dev 19: 2278–2283 gad.1324805 [pii];10.1101/gad.1324805 [doi]. 16166371 10.1101/gad.1324805 PMC1240035

[51] Ferguson AA, Springer MG, Fisher AL (2010) skn-1-Dependent and -independent regulation of aip-1 expression following metabolic stress in Caenorhabditis elegans. Mol Cell Biol 30: 2651–2667 MCB.01340-09;10.1128/MCB.01340-09. 20351174 10.1128/MCB.01340-09 PMC2876512

[52] Hasegawa K, Miwa S, Isomura K, Tsutsumiuchi K, Taniguchi H, et al (2008) Acrylamide-responsive genes in the nematode Caenorhabditis elegans. Toxicol Sci 101: 215–225 kfm276 [pii];10.1093/toxsci/kfm276 [doi]. 17989133 10.1093/toxsci/kfm276

[53] Snutch TP, Baillie DL (1983) Alterations in the pattern of gene expression following heat shock in the nematode Caenorhabditis elegans. Can J Biochem Cell Biol 61: 480–487. 6883176 10.1139/o83-064

[54] Gornati R, Monetti C, Vigetti D, Bosisio S, Fortaner S, et al (2002) Arsenic toxicity and HSP70 expression in Xenopus laevis embryos. Altern Lab Anim 30: 597–603. 12513685 10.1177/026119290203000606

[55] Das S, Pan D, Bera AK, Rana T, Bandyopadhyay S, et al (2010) Stress inducible heat shock protein 70: a potent molecular and toxicological signature in arsenic exposed broiler chickens. Mol Biol Rep 37: 3151–3155 10.1007/s11033-009-9894-7. 19826909 10.1007/s11033-009-9894-7

[56] Bernstam L, Nriagu J (2000) Molecular aspects of arsenic stress. J Toxicol Environ Health B Crit Rev 3: 293–322 10.1080/109374000436355 [doi]. 11055208 10.1080/109374000436355

[57] Shin H, Lee H, Fejes AP, Baillie DL, Koo HS, et al (2011) Gene expression profiling of oxidative stress response of C. elegans aging defective AMPK mutants using massively parallel transcriptome sequencing. BMC Res Notes 4: 34 1756-0500-4-34 [pii];10.1186/1756-0500-4-34 [doi]. 21303547 10.1186/1756-0500-4-34 PMC3045954

[58] Park SK, Tedesco PM, Johnson TE (2009) Oxidative stress and longevity in Caenorhabditis elegans as mediated by SKN-1. Aging Cell 8: 258–269 ACE473 [pii];10.1111/j.1474-9726.2009.00473.x [doi]. 19627265 10.1111/j.1474-9726.2009.00473.x PMC2762118

[59] Fukada T, Yamasaki S, Nishida K, Murakami M, Hirano T (2011) Zinc homeostasis and signaling in health and diseases: Zinc signaling. J Biol Inorg Chem 16: 1123–1134 10.1007/s00775-011-0797-4. 21660546 10.1007/s00775-011-0797-4 PMC3176402

[60] Boyer TD (1989) The glutathione S-transferases: an update. Hepatology 9: 486–496 S0270913989000601 [pii]. 2646197 10.1002/hep.1840090324

[61] Douglas KT (1987) Mechanism of action of glutathione-dependent enzymes. Adv Enzymol Relat Areas Mol Biol 59: 103–167. 2880477 10.1002/9780470123058.ch3

[62] Tocher DR, Leaver MJ, Hodgson PA (1998) Recent advances in the biochemistry and molecular biology of fatty acyl desaturases. Prog Lipid Res 37: 73–117 S0163-7827(98)00005-8 [pii]. 9829122 10.1016/s0163-7827(98)00005-8

[63] Richardson DR, Lok HC (2008) The nitric oxide-iron interplay in mammalian cells: transport and storage of dinitrosyl iron complexes. Biochim Biophys Acta 1780: 638–651 S0304-4165(07)00301-7;10.1016/j.bbagen.2007.12.009. 18206118 10.1016/j.bbagen.2007.12.009

[64] Lynn S, Gurr JR, Lai HT, Jan KY (2000) NADH oxidase activation is involved in arsenite-induced oxidative DNA damage in human vascular smooth muscle cells. Circ Res 86: 514–519. 10720412 10.1161/01.res.86.5.514

[65] Kim WK, Meliton V, Bourquard N, Hahn TJ, Parhami F (2010) Hedgehog signaling and osteogenic differentiation in multipotent bone marrow stromal cells are inhibited by oxidative stress. J Cell Biochem 111: 1199–1209 10.1002/jcb.22846. 20717924 10.1002/jcb.22846

[66] Dai RL, Zhu SY, Xia YP, Mao L, Mei YW, et al (2011) Sonic hedgehog protects cortical neurons against oxidative stress. Neurochem Res 36: 67–75 10.1007/s11064-010-0264-6. 20848190 10.1007/s11064-010-0264-6

[67] Koppenol WH (1993) The centennial of the Fenton reaction. Free Radic Biol Med 15: 645–651. 8138191 10.1016/0891-5849(93)90168-t

[68] Meneghini R (1997) Iron homeostasis, oxidative stress, and DNA damage. Free Radic Biol Med 23: 783–792 S0891-5849(97)00016-6 [pii]. 9296456 10.1016/s0891-5849(97)00016-6

[69] Ahmad S, Kitchin KT, Cullen WR (2000) Arsenic species that cause release of iron from ferritin and generation of activated oxygen. Arch Biochem Biophys 382: 195–202 S0003-9861(00)92023-X [pii];10.1006/abbi.2000.2023 [doi]. 11068869 10.1006/abbi.2000.2023

[70] Cairo G, Bardella L, Schiaffonati L, Arosio P, Levi S, et al (1985) Multiple mechanisms of iron-induced ferritin synthesis in HeLa cells. Biochem Biophys Res Commun 133: 314–321 0006-291X(85)91877-7 [pii]. 4074370 10.1016/0006-291x(85)91877-7

[71] Tsuji Y, Ayaki H, Whitman SP, Morrow CS, Torti SV, et al (2000) Coordinate transcriptional and translational regulation of ferritin in response to oxidative stress. Mol Cell Biol 20: 5818–5827. 10913165 10.1128/mcb.20.16.5818-5827.2000 PMC86059

[72] Wang W, Knovich MA, Coffman LG, Torti FM, Torti SV (2010) Serum ferritin: Past, present and future. Biochim Biophys Acta 1800: 760–769 S0304-4165(10)00085-1 [pii];10.1016/j.bbagen.2010.03.011 [doi]. 20304033 10.1016/j.bbagen.2010.03.011 PMC2893236

[73] Miotto G, Tessaro S, Rotta GA, Bonatto D (2007) In silico analyses of Fsf1 sequences, a new group of fungal proteins orthologous to the metazoan sideroblastic anemia-related sideroflexin family. Fungal Genet Biol 44: 740–753 S1087-1845(06)00234-9 [pii];10.1016/j.fgb.2006.12.004 [doi]. 17240176 10.1016/j.fgb.2006.12.004

[74] Robinson JF, Yu X, Moreira EG, Hong S, Faustman EM (2011) Arsenic- and cadmium-induced toxicogenomic response in mouse embryos undergoing neurulation. Toxicol Appl Pharmacol 250: 117–129 S0041-008X(10)00360-1 [pii];10.1016/j.taap.2010.09.018 [doi]. 20883709 10.1016/j.taap.2010.09.018 PMC3014392

[75] Nordstrom DK (2002) Public health. Worldwide occurrences of arsenic in ground water. Science 296: 2143–2145 10.1126/science.1072375 [doi];296/5576/2143 [pii]. 12077387 10.1126/science.1072375

[76] Menzel R, Swain SC, Hoess S, Claus E, Menzel S, et al (2009) Gene expression profiling to characterize sediment toxicity–a pilot study using Caenorhabditis elegans whole genome microarrays. BMC Genomics 10: 160 1471-2164-10-160 [pii];10.1186/1471-2164-10-160 [doi]. 19366437 10.1186/1471-2164-10-160 PMC2674462

[77] Greiner J, Ringhoffer M, Taniguchi M, Hauser T, Schmitt A, et al (2003) Characterization of several leukemia-associated antigens inducing humoral immune responses in acute and chronic myeloid leukemia. Int J Cancer 106: 224–231 10.1002/ijc.11200 [doi]. 12800198 10.1002/ijc.11200

[78] Okada H, Attanucci J, Giezeman-Smits KM, Brissette-Storkus C, Fellows WK, et al (2001) Immunization with an antigen identified by cytokine tumor vaccine-assisted SEREX (CAS) suppressed growth of the rat 9L glioma in vivo. Cancer Res 61: 2625–2631. 11289140

[79] Giezeman-Smits KM, Okada H, Brissette-Storkus CS, Villa LA, Attanucci J, et al (2000) Cytokine gene therapy of gliomas: induction of reactive CD4+ T cells by interleukin-4-transfected 9L gliosarcoma is essential for protective immunity. Cancer Res 60: 2449–2457. 10811123

[80] Resto VA, Caballero OL, Buta MR, Westra WH, Wu L, et al (2000) A putative oncogenic role for MPP11 in head and neck squamous cell cancer. Cancer Res 60: 5529–5535. 11034098

[81] Zhang X, Su Y, Zhang M, Sun Z (2012) Opposite effects of arsenic trioxide on the Nrf2 pathway in oral squamous cell carcinoma in vitro and in vivo. Cancer Lett 318: 93–98 S0304-3835(11)00742-7 [pii];10.1016/j.canlet.2011.12.005 [doi]. 22155346 10.1016/j.canlet.2011.12.005

[82] Sunde JS, Donninger H, Wu K, Johnson ME, Pestell RG, et al (2006) Expression profiling identifies altered expression of genes that contribute to the inhibition of transforming growth factor-beta signaling in ovarian cancer. Cancer Res 66: 8404–8412 66/17/8404 [pii];10.1158/0008-5472.CAN-06-0683 [doi]. 16951150 10.1158/0008-5472.CAN-06-0683

[83] Wu K, Li A, Rao M, Liu M, Dailey V, et al (2006) DACH1 is a cell fate determination factor that inhibits cyclin D1 and breast tumor growth. Mol Cell Biol 26: 7116–7129 26/19/7116 [pii];10.1128/MCB.00268-06 [doi]. 16980615 10.1128/MCB.00268-06 PMC1592900

[84] Wu K, Liu M, Li A, Donninger H, Rao M, et al (2007) Cell fate determination factor DACH1 inhibits c-Jun-induced contact-independent growth. Mol Biol Cell 18: 755–767 E06-09-0793 [pii];10.1091/mbc.E06-09-0793 [doi]. 17182846 10.1091/mbc.E06-09-0793 PMC1805093

[85] Popov VM, Zhou J, Shirley LA, Quong J, Yeow WS, et al (2009) The cell fate determination factor DACH1 is expressed in estrogen receptor-alpha-positive breast cancer and represses estrogen receptor-alpha signaling. Cancer Res 69: 5752–5760 69/14/5752 [pii];10.1158/0008-5472.CAN-08-3992 [doi]. 19605405 10.1158/0008-5472.CAN-08-3992 PMC3244171

[86] Popov VM, Wu K, Zhou J, Powell MJ, Mardon G, et al (2010) The Dachshund gene in development and hormone-responsive tumorigenesis. Trends Endocrinol Metab 21: 41–49 S1043-2760(09)00145-3 [pii];10.1016/j.tem.2009.08.002 [doi]. 19896866 10.1016/j.tem.2009.08.002 PMC2818438

[87] Tenen DG (2001) Abnormalities of the CEBP alpha transcription factor: a major target in acute myeloid leukemia. Leukemia 15: 688–689. 11368383 10.1038/sj.leu.2402088

[88] Mangelsdorf DJ, Thummel C, Beato M, Herrlich P, Schutz G, et al (1995) The nuclear receptor superfamily: the second decade. Cell 83: 835–839 0092-8674(95)90199-X [pii]. 8521507 10.1016/0092-8674(95)90199-x PMC6159888

[89] Knudsen KE, Penning TM (2010) Partners in crime: deregulation of AR activity and androgen synthesis in prostate cancer. Trends Endocrinol Metab 21: 315–324 S1043-2760(10)00004-4 [pii];10.1016/j.tem.2010.01.002 [doi]. 20138542 10.1016/j.tem.2010.01.002 PMC2862880

[90] de Bono JS, Molife LR, Sonpavde G, Maroto JP, Calvo E, et al (2012) Phase II study of eribulin mesylate (E7389) in patients with metastatic castration-resistant prostate cancer stratified by prior taxane therapy. Ann Oncol 23: 1241–1249 mdr380 [pii];10.1093/annonc/mdr380 [doi]. 21903605 10.1093/annonc/mdr380

