# Supplementary figures and images for: Genomic Analysis of Stress Response against Arsenic in Caenorhabditis elegans
Source: PLoS One. 2013 Jul 24;8(7):e66431. doi: 10.1371/journal.pone.0066431 (PMC3722197; doi:10.1371/journal.pone.0066431)

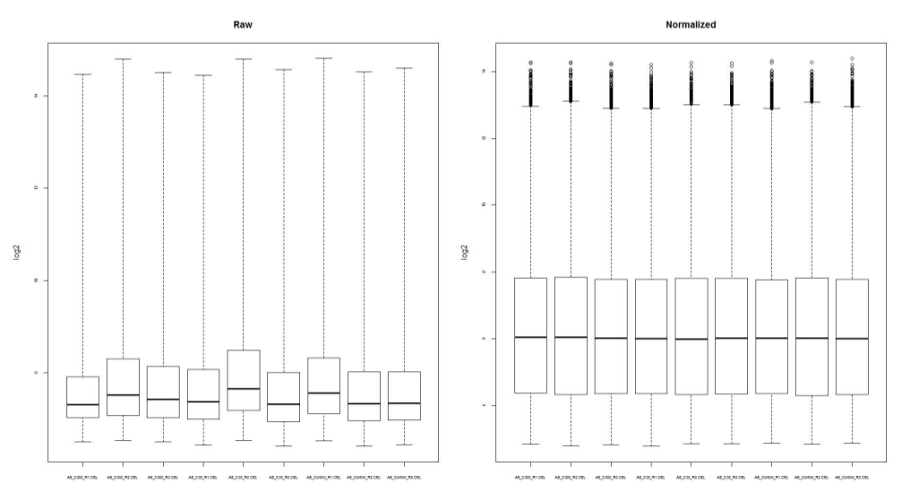

Supplement: Figure S1 — Boxplots which depict A) row data, and B) data after normalization. PM only probe set signal was applied. (TIF) [file pone.0066431.s001.tif]

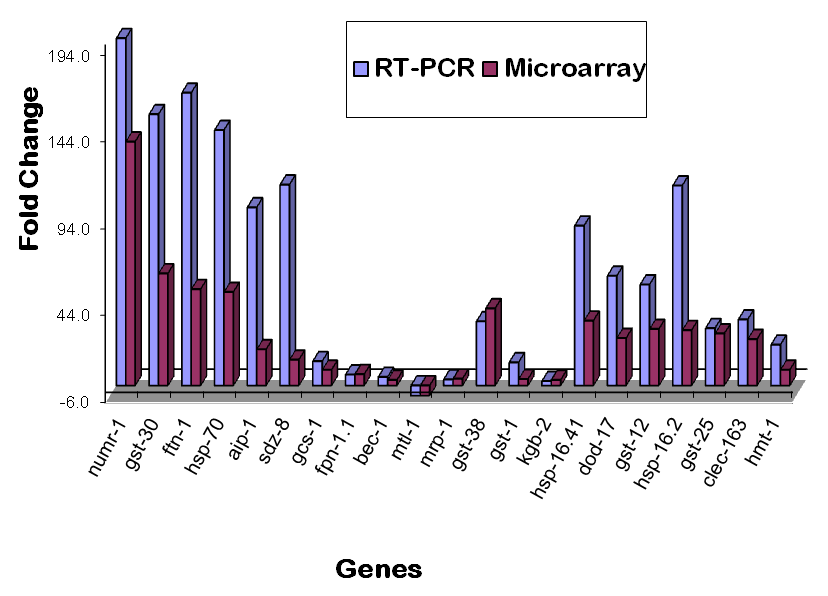

Supplement: Figure S2 — qRT-PCR results for selected high ranker genes. (TIF) [file pone.0066431.s002.tif]

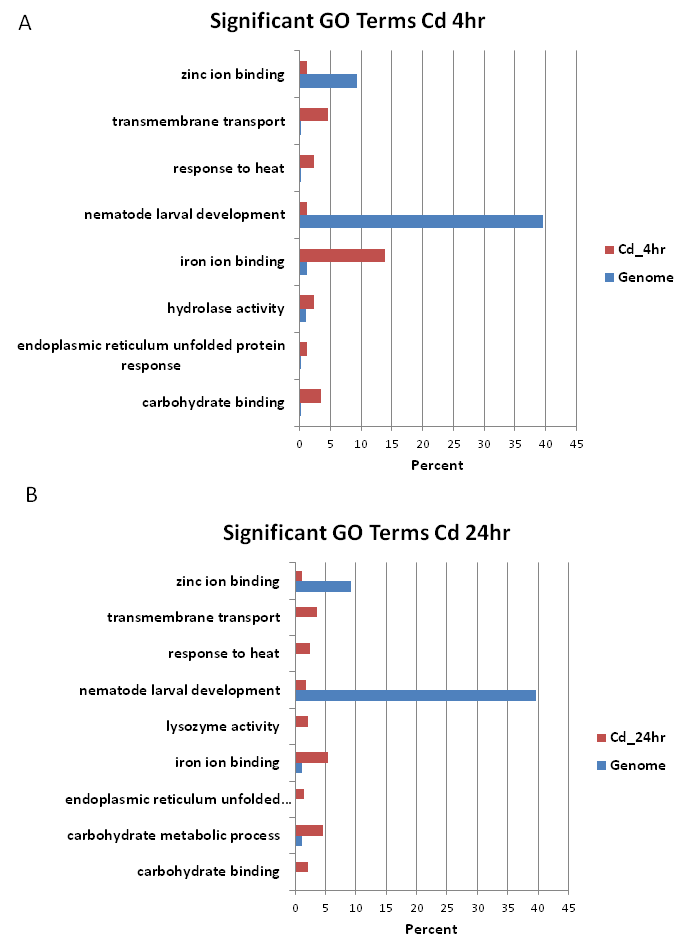

Supplement: Figure S3 — GO term enrichments with cadmium in C. elegans . (A) Enrichment of GO categories for 4-hour cadmium exposure. (B) Enrichment of GO categories for 24-hour cadmium exposure. (TIF) [file pone.0066431.s003.tif]

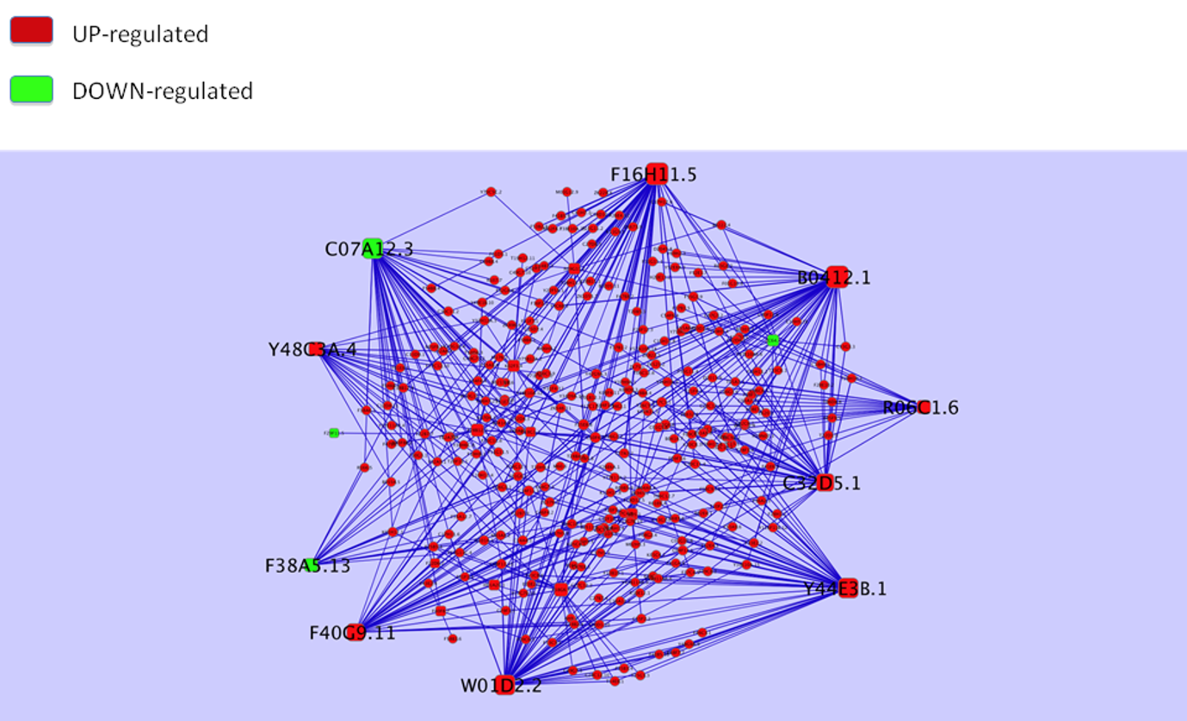

Supplement: Figure S4 — Significant transcription factor- gene interactions in high arsenic condition. The color of the nodes represent the overall expression of the gene (green: down-regulated, red: up-regulated). The size of vertices is proportional to their degree (i.e., number of edges incident on them). Each node is labeled with the corresponding gene or TF's name. Rounded squares represent transcription factors, and circles represent putative target genes of these transcription factors. The layout of the network was generated manually on Cytoscape. (TIF) [file pone.0066431.s004.tif]
