# Supplementary material for: Genomic Analysis of Stress Response against Arsenic in Caenorhabditis elegans
Source: PLoS One. 2013 Jul 24;8(7):e66431. doi: 10.1371/journal.pone.0066431 (PMC3722197; doi:10.1371/journal.pone.0066431)
Supplement: Table S1 — List of primers used for qRT-PCR. (DOCX) [file pone.0066431.s005.docx]

Table S1: List of primers used for qRT-PCR

| Primers | Sequences | Sources |
| --- | --- | --- |
| *numr-1 (F)* | ggaagacgaggaatggatca | This study |
| *numr-1 (R)* | tgcgttcaaatggatgttgt | This study |
| *gst-30 (F)* | gggcaaaagtttgaggatca | This study |
| *gst-30 (R)* | tcttcccatggacatcttcc | This study |
| *ftn-1 (F)* | gacgcgcacttgacaaatta | This study |
| *ftn-1 (R)* | ccggctctcttgatatttgc | This study |
| *hsp-70 (F)* | cttggttgggggatcaact | This study |
| *hsp-70 (R)* | ccaaaggctactgcttcgtc | This study |
| *aip-1 (F)* | ccaaatctcggaaagcactg | This study |
| *aip-1 (R)* | ttgagccagtcggacaatta | This study |
| *sdz-8 (F)* | gcaactgctagagacgttgaga | This study |
| *sdz-8 (R)* | ttctccaactttcgacacaaaa | This study |
| *gcs-1 (F)* | aaccgtaccgcacattgatt | This study |
| *gcs-1 (R)* | ccccatttcaattgatctcc | This study |
| *fpn-1.1 (F)* | cggcatttcttctcaccact | This study |
| *fpn-1.1 (R)* | acccatgtcattcctccaag | This study |
| *bec-1 (F)* | caaagaaggccagattcagc | This study |
| *bec-1 (R)* | ctggagcatcagatgggaat | This study |
| *mtl-1 (F)* | ccagctggatgtaagggaga | This study |
| *mtl-1 (R)* | agcagttccctggtgttgat | This study |
| *mrp-1 (F)* | gttcgtgaactggtgtgtgg | This study |
| *mrp-1 (R)* | gtgctggccacaatttgtta | This study |
| *gst-38 (F)* | taccgatgaggagtgggaga | This study |
| *gst-38 (R)* | ctacctcgagcattggaagc | This study |
| *gst-1 (F)* | aagcaagttgcctacgagga | This study |
| *gst-1 (R)* | tctccggatagaagacatgga | This study |
| *kgb-2 (F)* | ccaacccgttatgtcaacct | This study |
| *kgb-2 (R)* | gattgctacgcgttgtgttg | This study |
| *hsp-16.41 (F)* | tcttggacgaactcactgga | This study |
| *hsp-16.41 (R)* | gacatcgagttgaaccgaaaa | This study |
| *dod-17 (F)* | cgtttgctactgcaatttcg | This study |
| *dod-17 (R)* | aggctgagtctcgttccaag | This study |
| *gst-12 (F)* | ggagttccgtttgaggatga | This study |
| *gst-12 (R)* | cgacgtttaggacaggcatt | This study |
| *hsp-16.2 (F)* | ctctgatggaacgccaattt | This study |
| *hsp-16.2 (R)* | cgttgagattgatggcaaac | This study |
| *gst-25 (F)* | gtgagccgattcgtatgctt | This study |
| *gst-25 (R)* | cccagattgcacaatttcct | This study |
| *clec-163 (F)* | tgacggcgatgtttcttcta | This study |
| *clec-163 (R)* | caacaatgtcgagccaaaga | This study |
| *hmt-1 (F)* | aatgtcgagccgaaggacta | This study |
| *hmt-1 (R)* | ttgacgacgatgaagaatgtg | This study |
