# Supplementary material for: Genomic Analysis of Stress Response against Arsenic in Caenorhabditis elegans
Source: PLoS One. 2013 Jul 24;8(7):e66431. doi: 10.1371/journal.pone.0066431 (PMC3722197; doi:10.1371/journal.pone.0066431)
Supplement: Table S2 — List of genes differentially expressed in both high and low dose arsenic exposure (+/−1.5 fold). (DOCX) [file pone.0066431.s006.docx]

Table S2: List of genes differentially expressed in both high and low dose arsenic exposure (+/- 1.5 fold).

| **Gene Name** | **Brief Description** |
| --- | --- |
| *aat-7* | predicted amino acid transporter catalytic subunit |
| *ads-1* | ortholog of human alkyl-dihydroxyacetonephosphate synthase precursor (AGPS) that is required for normal larval development |
| *aip-1* | AN-1-like zinc finger-containing protein homologous to arsenite-inducible RNA-associated protein (AIRAP) |
| *air-2* | aurora/Ipl1-related serine/threonine protein kinase |
| *alh-10* | aldehyde dehydrogenase |
| *amt-4* | member of the ammonium transporter protein family |
| *aqp-1* | aquaglyceroporin |
| *aqp-11* | putative aquaporin |
| *asm-2* | similar to human acid sphingomyelinase (ASM) |
| *bec-1* | orthologous to the yeast and mammalian autophagy proteins Apg6/Vps30p/beclin1 |
| *cat-4* | ortholog of the human GTP cyclohydrolase I gene |
| *cdd-1* | cytidine deaminase |
| *cdk-1* | cyclin-dependent kinase |
| *cpg-1* | encodes a protein with three chitin-binding peritrophin-A domains and two mucin-like regions |
| *cey-2* | cold-shock/Y-box domain-containing gene |
| *cln-3.3* | predicted transmembrane protein that comprises one of three C. elegans homologs of human CLN3 |
| *cnc-4* | caenacin peptide |
| *col-8* | collagen |
| *col-19* | collagen |
| *col-62* | collagen |
| *col-76* | collagen |
| *col-103* | collagen |
| *col-143* | collagen |
| *col-178* | collagen |
| *col-184* | collagen |
| *cpr-4* | cathepsin B-like cysteine protease gene |
| *cpr-5* | cysteine protease. |
| *ctl-2* | catalase |
| *cyb-2.2* | cyclin |
| *daf-21* | member of the Hsp90 family of molecular chaperones |
| *dao-3* | encodes a protein containing tetrahydrofolate dehydrogenase/cyclohydrolase catalytic and NAD(P)-binding domains |
| *dhs-2* | short-chain dehydrogenase predicted to be mitochondrial. |
| *dhs-4* | short-chain dehydrogenase predicted to be mitochondrial. |
| *dhs-8* | short-chain dehydrogenase predicted to be mitochondrial. |
| *dhs-9* | member of the short-chain dehydrogenases/reductases family (SDR). |
| *dhs-12* | alcohol dehydrogenase |
| *dhs-20* | short-chain dehydrogenase predicted to be mitochondrial. |
| *dhs-22* | short-chain dehydrogenase predicted to be mitochondrial. |
| *dlk-1* | mitogen-activated protein kinase kinase kinase (MAPKKK) |
| *ech-7* | enoyl-CoA hydratase |
| *ech-9* | enoyl-CoA hydratase |
| *elo-1* | a component of C-18 polyunsaturated fatty acid (PUFA) elongase |
| *elo-5* | polyunsaturated fatty acid (PUFA) elongase |
| *elo-6* | polyunsaturated fatty acid (PUFA) elongase |
| *fis-2* | unknown |
| *ftn-1* | ferritin heavy chain homolog |
| *ftn-2* | ferritin heavy chain homolog |
| *gcs-1* | gamma-glutamine cysteine synthetase heavy chain (GCS(h)) |
| *gfi-1* | encodes a protein that contains 21 ET modules |
| *gln-5* | carbamoyl-phosphate synthase (CPSase) (2 domains), Biotin-requiring enzymes |
| *gln-6* | glutamine synthase |
| *gna-2* | glucosamine 6-phosphate N-acetyltransferase |
| *grl-14* | hedgehog-like protein |
| *gst-1* | glutathione S-transferase |
| *gst-2* | glutathione S-transferase |
| *gst-4* | glutathione S-transferase |
| *gst-5* | glutathione S-transferase |
| *gst-6* | glutathione S-transferase |
| *gst-7* | glutathione S-transferase |
| *gst-8* | glutathione S-transferase |
| *gst-10* | glutathione S-transferase |
| *gst-12* | glutathione S-transferase |
| *gst-13* | glutathione S-transferase |
| *gst-14* | glutathione S-transferase |
| *gst-16* | glutathione S-transferase |
| *gst-19* | glutathione S-transferase |
| *gst-20* | glutathione S-transferase |
| *gst-22* | glutathione S-transferase |
| *gst-25* | glutathione S-transferase |
| *gst-27* | glutathione S-transferase |
| *gst-30* | glutathione S-transferase |
| *gst-31* | glutathione S-transferase |
| *gst-38* | glutathione S-transferase |
| *gst-39* | glutathione S-transferase |
| *gst-40* | glutathione S-transferase |
| *hmt-1* | encodes a predicted transmembrane half-molecule ATP-binding cassette (ABC) transporter |
| *hgo-1* | homogentisate 1,2-dioxygenase |
| *hil-3* | histone h1 |
| *hsp-16.1* | hsp16/hsp20/alphaB-crystallin (HSP16) family |
| *hsp-16.2* | hsp16/hsp20/alphaB-crystallin (HSP16) family |
| *hsp-16.41* | hsp16/hsp20/alphaB-crystallin (HSP16) family |
| *hsp-16.49* | hsp16/hsp20/alphaB-crystallin (HSP16) family |
| *hsp-17* | hsp16/hsp20/alphaB-crystallin (HSP16) family |
| *hsp-70* | hsp70 family of molecular chaperones. |
| *ida-1* | protein tyrosine phosphatase-like receptor |
| *ife-3* | homolog of the mRNA cap-binding protein eIF4E |
| *ins-35* | insulin-related peptide |
| *inx-15* | unknown |
| *itr-1* | putative inositol (1,4,5) trisphosphate receptor |
| *kgb-2* | protein kinase |
| *lec-8* | lectin |
| *lec-9* | lectin |
| *lec-10* | galactose-binding lectin |
| *lec-11* | galectin family |
| *lys-4* | lysozyme |
| *lys-8* | lysozyme |
| *max-1* | novel, conserved PH/MyTH4/FERM domain-containing gene |
| *mbf-1* | Helix-turn-helix motif containing gene |
| *mel-32* | serine hydroxymethyltransferase |
| *mrp-1* | ATP-binding cassette (ABC) transporter |
| *mrp-7* | ATP-binding cassette (ABC) transporter |
| *msp-31* | major sperm protein family |
| *msp-33* | major sperm protein family |
| *msp-49* | major sperm protein family |
| *mtl-1* | metallothionein |
| *mtl-2* | metallothionein |
| *nas-31* | zinc metalloprotease |
| *nhr-28* | nuclear receptor superfamily |
| *nhr-34* | nuclear receptor superfamily |
| *nhr-55* | nuclear receptor superfamily |
| *nhr-70* | nuclear receptor superfamily |
| *nhr-74* | nuclear receptor superfamily |
| *nhr-104* | nuclear receptor superfamily |
| *nhr-115* | nuclear receptor superfamily |
| *nhx-2* | sodium/proton exchanger |
| *nlp-31* | neuropeptide-like protein |
| *nlt-1* | SCP-2 sterol transfer family |
| *pas-1* | type 6 alpha subunit of the 26S proteasome's 20S protease core particle (CP) |
| *pbs-3* | B-type subunit of the 26S proteasome's 20S protease core particle |
| *pbs-6* | homolog of mammalian PSMB1 |
| *pcp-2* | lysosomal carboxypeptidase |
| *pcp-4* | peptidase |
| *pgp-1* | member of the P-glycoprotein subclass of the ATP-binding cassette (ABC) transporter superfamily |
| *pos-1* | CCCH-type zinc-finger protein |
| *prk-1* | Protein kinase |
| *rme-2* | low-density lipoprotein (LDL) receptor superfamily |
| *rpn-10* | member of the von Willebrand factor, type A superfamily |
| *rpn-11* | non-ATPase subunit of the 19S regulatory complex of the proteasome |
| *rpt-2* | ATPase |
| *rpt-6* | triple A ATPase that is a subunit of the 26S proteasome 19S regulatory particle (RP) base subcomplex |
| *sax-1* | homolog of Ndr kinase |
| *skr-19* | homolog of Skp1 |
| *spe-11* | novel, conserved PH/MyTH4/FERM domain-containing gene |
| *spn-4* | encodes a protein containing an RNP-type RNA-binding domain |
| *srr-4* | unknown |
| *ssp-9* | unknown |
| *sss-2* | unknown |
| *nck-1* | SH2/SH3 domain-containing protein |
| *prdx-2* | 2-Cys peroxiredoxins |
| *tba-7* | tubulin alpha subunit |
| *tbb-6* | tubulin beta chain |
| *tsp-10* | transpanin |
| *ubc-20* | ubiquitin conjugating enzyme |
| *vit-1* | vitellogenin |
| *vit-3* | vitellogenin |
| *vit-4* | vitellogenin |
| *vit-5* | vitellogenin |
| *vit-6* | vitellogenin |
| *gspd-1* | orthologous to human GLUCOSE-6-PHOSPHATE DEHYDROGENASE |
| *B0272.4* | enoyl-CoA hydratase/isomerase |
| *clec-41* | C-type lectin |
| *B0513.4* | unknown |
| *C01H6.4* | Flavin-binding monooxygenase-like |
| *C05C10.5* | novel |
| *C07A9.8* | Putative membrane protein encoding gene |
| *C08E8.4* | unknown |
| *fbxa-98* | F-box motif containing gene |
| *ugt-22* | UDP-glucoronosyl and UDP-glucosyl transferases |
| *C10C5.2* | unknown |
| *wht-2* | subfamily G of the ATP-binding cassette transporters |
| *C12C8.2* | cystathionine gamma-lyase orthologous to human CTH |
| *C13B4.1* | unknown |
| *C14B1.2* | unknown |
| *C15A11.4* | amine oxidase |
| *C15A11.7* | unknown |
| *C15H11.8* | DNA-directed RNA polymerase I like |
| *clec-87* | C-type lectin |
| *C28D4.5* | unknown |
| *C29F3.7* | unknown |
| *C32H11.1* | unknown |
| *C32H11.3* | unknown |
| *C32H11.4* | unknown |
| *dod-24* | unknown |
| *C35A5.3* | sodium/phosphate transport protein |
| *cyp-25A1* | cytochrome P450 |
| *cyp-25A2* | cytochrome P450 |
| *C39E9.12* | unknown |
| *C40H1.7* | unknown |
| *cdc-48.2* | encodes a AAA (ATPase associated with various cellular activities) |
