# Supplementary material for: Genomic Analysis of Stress Response against Arsenic in Caenorhabditis elegans
Source: PLoS One. 2013 Jul 24;8(7):e66431. doi: 10.1371/journal.pone.0066431 (PMC3722197; doi:10.1371/journal.pone.0066431)
Supplement: Table S3 — List of genes differentially expressed in both, high dose arsenic, and cadmium 24-hour exposures (+/−1.5 fold). (DOCX) [file pone.0066431.s007.docx]

Table S3: List of genes differentially expressed in both, high dose arsenic, and cadmium 24-hour exposures (+/- 1.5 fold).

| **Gene Name** | **Brief Description** |
| --- | --- |
| *acdh-2* | short-chain acyl-CoA dehydrogenase |
| *alh-5* | aldehyde dehydrogenase |
| *asm-2* | sphingomyelin phosphodiesterase |
| *B0284.2* | unknown |
| *C06E4.3* | dehydrogenase |
| *C08E3.1* | unknown |
| *C08E8.4* | unknown |
| *C10C5.2* | unknown |
| *C15C8.3* | cathepsin-like protease |
| *C18A11.1* | unknown |
| *C23H5.8* | unknown |
| *C24B9.3* | unknown |
| *C25H3.10* | unknown |
| *C27H5.4* | unknown |
| *C29F3.7* | unknown |
| *C32F10.4* | unknown |
| *C48B4.1* | Acyl-CoA oxidase I |
| *C50F7.5* | unknown |
| *cdr-1* | cadmium-inducible lysosomal family |
| *clec-15* | C-type lectin |
| *clec-47* | C-type lectin |
| *clec-51* | C-type lectin |
| *clec-61* | C-type lectin |
| *clec-70* | C-type lectin |
| *clec-82* | C-type lectin |
| *col-120* | unknown |
| *cpr-2* | cysteine protease |
| *cyp-13A5* | cytochrome P450 |
| *cyp-33C7* | cytochrome P451 |
| *D1025.2* | unknown |
| *F01D5.5* | unknown |
| *F02A9.1* | unknown |
| *F08A8.2* | Acyl-Coenzyme A Oxidase |
| *F08A8.3* | Acyl-Coenzyme A Oxidase |
| *F12B6.2* | sodium dependent-phosphate transporter |
| *F13H6.3* | carboxylesterase |
| *F15B9.6* | phospholipase A2 |
| *F15E11.1* | unknown |
| *F15E11.12* | unknown |
| *F15E11.15* | unknown |
| *F18E2.1* | acid phophatase like |
| *F19C7.2* | lysosomal carboxypeptidase |
| *F19C7.4* | lysosomal carboxypeptidase |
| *F21C10.9* | unknown |
| *F22B7.9* | unknown |
| *F28A12.4* | peptidase |
| *F35E12.8* | unknown |
| *F42C5.3* | unknown |
| *F44E5.4* | Heat shock hsp70 |
| *F44G3.2* | arginine kinase |
| *F45D11.1* | unknown |
| *F47H4.2* | F-box motif containing protein |
| *F49F1.6* | signal sequence and several ShK toxin domains containing gene |
| *F52E1.5* | unknown |
| *F53B2.8* | unknown |
| *F54B8.4* | homolog of Death Associated Protein 1 (DAP-1) |
| *F55G11.4* | unknown |
| *F56C3.9* | unknown |
| *F58E6.4* | unknown |
| *F59B1.8* | unknown |
| *F59D6.3* | aspartyl protease |
| *far-3* | O.volvulus antigen peptide like |
| *gst-19* | glutathione S-transferase |
| *gst-38* | glutathione S-transferase |
| *gst-9* | glutathione S-transferase |
| *H06H21.8* | unknown |
| *hsp-16.2* | heat shock protein |
| *hsp-16.41* | heat shock protein |
| *hsp-17* | heat shock protein |
| *hsp-70* | heat shock protein |
| *ilys-3* | unknown |
| *ins-7* | insulin/IGF-1-like peptide |
| *K04F1.9* | unknown |
| *K09D9.1* | unknown |
| *K10B2.2* | Carboxypeptidase |
| *lys-4* | unknown |
| *lys-5* | unknown |
| *lys-6* | unknown |
| *M01G12.9* | unknown |
| *mrp-3* | multidrug resistance-associated protein |
| *mtl-1* | metallothionein |
| *mtl-2* | metallothionein |
| *mxl-3* | Helix-loop-helix DNA-binding domain |
| *nhr-206* | nuclear hormone receptor |
| *nit-1* | Nitrilase |
| *npa-1* | lipid carrier protein encoding gene |
| *PDB1.1* | unknown |
| *pgp-1* | multidrug resistance protein |
| *pmp-5* | ABC transporter |
| *rrf-2* | RNA-directed RNA polymerase (RdRP) homolog |
| *spp-2* | orthologous to the human gene INTERFERON GAMMA RECEPTOR 2 (IFNGR2) |
| *T01D3.6* | EGF-like domains |
| *T04A8.5* | Amidophosphoribosyltransferase |
| *T07C12.9* | N-methyltransferase |
| *T12D8.5* | unknown |
| *T15B7.1* | unknown |
| *T16G1.6* | unknown |
| *T22B7.7* | unknown |
| *T24B8.5* | ShK-like toxin |
| *T26F2.2* | unknown |
| *T28A11.19* | unknown |
| *T28D9.3* | unknown |
| *thn-1* | Arabidopsis pathogenesis-related protein 5 like |
| *thn-2* | Arabidopsis pathogenesis-related protein 5 like |
| *ugt-16* | UDP-glucuronosyltransferase |
| *ugt-2* | UDP-glucuronosyltransferase |
| *ugt-31* | 7TM chemoreceptor, ugt family |
| *ugt-41* | UGT family |
| *ugt-62* | UDP-glucuronosyltransferase |
| *vit-1* | unknown |
| *W01A11.1* | epoxide hydrolase |
| *W03G1.5* | unknown |
| *W09G12.7* | unknown |
| *Y105C5A.12* | unknown |
| *Y19D10B.7* | unknown |
| *Y39B6A.24* | unknown |
| *Y39G8B.7* | unknown |
| *Y43F8A.3* | unknown |
| *Y45F10D.6* | unknown |
| *Y48E1B.8* | unknown |
| *Y4C6B.6* | homolog of the human gene GLCM, which when mutated leads to Gaucher disease type I |
| *Y51A2B.1* | unknown |
| *Y58A7A.5* | unknown |
| *Y6E2A.5* | unknown |
| *ZC204.12* | unknown |
| *ZK742.3* | unknown |
