# Supplementary material for: Genomic Analysis of Stress Response against Arsenic in Caenorhabditis elegans
Source: PLoS One. 2013 Jul 24;8(7):e66431. doi: 10.1371/journal.pone.0066431 (PMC3722197; doi:10.1371/journal.pone.0066431)
Supplement: Table S4 — List of genes differentially expressed in both, high dose arsenic, and paraquat exposures (+/−1.5 fold). (DOCX) [file pone.0066431.s008.docx]

Table S4: List of genes differentially expressed in both, high dose arsenic, and paraquat exposures (+/- 1.5 fold).

| **Gene Name** | **Brief Description** |
| --- | --- |
| *acbp-1* | acyl-CoA-binding protein |
| *aex-5* | neuroendocrine convertase 2 precursor like |
| *alp-1* | LIM domains |
| *arf-1.2* | ADP-ribosylation factor homolog |
| *arf-3* | ADP-ribosylation factor homolog |
| *atp-4* | ATP synthase subunit |
| *bli-1* | collagen |
| *bli-2* | collagen |
| *C04F12.7* | unknown |
| *C08E3.1* | unknown |
| *C08E3.13* | unknown |
| *C14C11.7* | unknown |
| *C17H12.8* | unknown |
| *C18B2.5* | unknown |
| *C18E9.4* | unknown |
| *C28H8.4* | ER lumen protein retaining receptor |
| *C30G12.2* | Alcohol dehydrogenase |
| *C33A12.1* | NADH-ubiquinone oxidoreductase 13 KD-B subunit |
| *C37A2.8* | unknown |
| *C44B7.10* | unknown |
| *cct-5* | TCP-1 like chaperonin |
| *clec-1* | clec family, C-type lectin |
| *clec-85* | clec family, C-type lectin |
| *clic-1* | clathrin light chain |
| *col-120* | collagen |
| *col-130* | collagen |
| *col-166* | collagen |
| *col-167* | collagen |
| *col-168* | collagen |
| *col-176* | collagen |
| *col-180* | collagen |
| *col-38* | collagen |
| *col-41* | collagen |
| *col-49* | collagen |
| *col-77* | collagen |
| *cpg-8* | chondroitin proteoglycan |
| *cpg-9* | chondroitin proteoglycan |
| *cpi-1* | protease inhibitor |
| *cpl-1* | cathepsin-like protease |
| *cpr-1* | cathepsin-like cysteine protease |
| *dao-2* | unknown |
| *dao-4* | unknown |
| *ddp-1* | human deafness/dystonia peptide ortholog |
| *dim-1* | novel protein containing immunoglobulin like repeats |
| *dlc-1* | dynein light chain |
| *dnj-12* | DnaJ domain protein |
| *dpy-11* | thioredoxin |
| *dpy-5* | cuticular collagen |
| *elo-5* | integral membrane protein |
| *erm-1* | membrane protein |
| *F09E5.3* | Deoxyribose-phosphate aldolase |
| *F15B9.8* | Thrombospondin type 1 domain |
| *F25B5.3* | unknown |
| *F26E4.6* | cytochrome C oxidase |
| *F29B9.11* | unknown |
| *F29C4.2* | unknown |
| *F32A5.4* | Immunodominant antigen |
| *F33D4.6* | unknown |
| *F39H11.1* | Yeast YB81 protein like |
| *F42A10.7* | unknown |
| *F44E5.1* | unknown |
| *F47G9.1* | Fugu S31III125 protein |
| *F49C12.11* | unknown |
| *F49E2.5* | unknown |
| *F49H12.5* | unknown |
| *F52A8.5* | glycine cleavage system H protein like |
| *F53A9.1* | unknown |
| *F53A9.8* | unknown |
| *F53F1.4* | cuticlin |
| *F56A8.3* | Leucine Rich Repeat |
| *F56D3.1* | unknown |
| *F57B10.5* | unknown |
| *F57H12.6* | unknown |
| *F58F12.1* | ATP synthase |
| *fkb-5* | peptidyl prolyl cis-trans isomerase |
| *ftn-2* | ferritin |
| *glh-1* | RNA helicase |
| *grd-5* | hedgehog-like protein |
| *grl-4* | hedgehog-like protein |
| *grl-7* | hedgehog-like protein |
| *gst-27* | glutathione S-transferase |
| *H28O16.1* | ATP synthase Alpha chain |
| *H42K12.3* | unknown |
| *hsp-16.1* | heat shock protein |
| *hsp-16.2* | heat shock protein |
| *hsp-16.41* | heat shock protein |
| *hsp-16.49* | heat shock protein |
| *hsp-25* | heat shock protein |
| *hsp-3* | heat shock protein |
| *hsp-6* | heat shock protein |
| *ife-3* | translation initiation factor |
| *ile-1* | P58 protein like |
| *K01D12.9* | unknown |
| *K08D12.3* | unknown |
| *K09G1.1* | unknown |
| *K10C2.3* | aspartyl protease |
| *K12H4.5* | unknown |
| *lbp-6* | fatty acid-binding protein |
| *lec-6* | lectin |
| *lev-11* | tropomyosin |
| *lgg-1* | Atg8p/ LC3 |
| *lsm-5* | small nuclear ribonucleoprotein E (SNRNP-E) like |
| *lsm-6* | U6 small nuclear RNA-associated Sm-like protein LSm6 |
| *M02H5.8* | unknown |
| *mai-2* | ATPase inhibitor |
| *mca-3* | membrane calcium ATPase |
| *mdh-1* | lactate dehydrogenase |
| *mdt-28* | metazoan-specific subunit of the Mediator transcriptional regulatory complex |
| *mev-1* | Succinate dehydrogenase cytochrome b chain |
| *mlt-8* | unknown |
| *mtl-2* | metallothionein |
| *nlp-33* | neuropeptide like protein |
| *nuo-4* | NADH dehydrogenase |
| *oig-2* | one IG-domain protein |
| *pas-3* | endopeptidase |
| *pas-5* | proteasome zeta chain |
| *phb-2* | prohibitin |
| *pqn-32* | glutamine/asparagine (Q/N)-rich ('prion') domain protein |
| *qua-1* | hedgehog-like protein |
| *R02E4.3* | unknown |
| *R05H10.5* | Glutathione peroxidases |
| *R06C1.4* | RNA recognition motif. (aka RRM, RBD, or RNP domain) |
| *R07E5.13* | homolog of mammalian BRAIN PROTEIN 44-LIKE (BRP44L) |
| *R102.2* | unknown |
| *R10H10.3* | CUB domain, von Willebrand factor type A domain |
| *rab-5* | RAS-related protein |
| *ran-4* | nuclear transport factor 2 like |
| *rol-8* | Cutical collagen 6, col-6 |
| *rpl-24.2* | ribosomal protein |
| *rpt-4* | predicted ATPase subunit of the 19S regulatory complex of the proteasome |
| *rsp-3* | splicing factor |
| *sdhd-1* | orthologous to the human gene SUCCINATE DEHYDROGENASE COMPLEX, SUBUNIT D |
| *sgt-1* | TPR domain repeats |
| *skr-1* | cyclin A/CDK2-associated protein P19 like |
| *spp-23* | SaPosin like protein family |
| *spp-3* | SaPosin like protein family |
| *sqt-1* | cuticle collagen SQT-1 |
| *sym-1* | Drosophila chaoptin protein like |
| *T02H6.11* | ubiquinol-cytochrome c reductase complex subunit |
| *T07A9.9* | unknown |
| *T19C3.2* | unknown |
| *T23G7.3* | unknown |
| *T24B8.5* | ShK-like toxin peptide |
| *T26E3.4* | unknown |
| *T27F7.1* | unknown |
| *tag-18* | unknown |
| *tin-9.1* | Tin9 family of mitochondrial import protein |
| *tomm-7* | Tom7 family of mitochondrial import protein |
| *tsn-1* | human 100 kDa activator protein |
| *ubc-20* | ubiquitin conjugating enzyme |
| *unc-116* | Kinesin heavy chain |
| *vha-1* | ATP synthase subunit C (2 domains) |
| *vha-10* | vacuolar ATPase G subunit |
| *vha-11* | ortholog of subunit C of the cytoplasmic (V1) domain of vacuolar proton-translocating ATPase (V-ATPase) |
| *vha-15* | ATPase subunit |
| *vha-2* | Vacuolar ATP synthase subunit |
| *vha-4* | proteolipid protein PPA1 like protein |
| *vha-8* | ATPase |
| *W02D3.1* | cytochrome B5 |
| *W06A7.4* | unknown |
| *wrt-10* | hedgehog-like protein |
| *wrt-4* | hedgehog-like protein |
| *Y105C5A.12* | unknown |
| *Y105E8A.11* | unknown |
| *Y39A1A.7* | unknown |
| *Y44E3A.3* | unknown |
| *Y63D3A.7* | ortholog of the NDUFA2/B8 subunit of the mitochondrial NADH dehydrogenase (ubiquinone) complex (complex I) |
| *Y69A2AR.28* | unknown |
| *Y69A2AR.3* | unknown |
| *Y94H6A.10* | unknown |
| *ZC395.10* | unknown |
| *zip-2* | bZIP transcription factor |
| *ZK105.1* | unknown |
| *ZK1307.8* | protein kinase C substrate |
