# Supplementary material for: Genomic Analysis of Stress Response against Arsenic in Caenorhabditis elegans
Source: PLoS One. 2013 Jul 24;8(7):e66431. doi: 10.1371/journal.pone.0066431 (PMC3722197; doi:10.1371/journal.pone.0066431)
Supplement: Table S5 — List of genes differentially expressed in both, high dose arsenic, and hyperbaric oxygen exposures (+/−1.5 fold). (DOCX) [file pone.0066431.s009.docx]

Table S5: List of genes differentially expressed in both, high dose arsenic, and hyperbaric oxygen exposures (+/- 1.5 fold).

| ***Gene Name*** | **Brief Description** |
| --- | --- |
| *alh-9* | aldehyde dehydrogenase |
| *B0416.7* | unknown |
| *C02F5.7* | F-box protein |
| *C05B5.8* | unknown |
| *C05D9.9* | unknown |
| *C06B3.7* | unknown |
| *C06H5.7* | unknown |
| *C07A12.7* | unknown |
| *C08F11.13* | unknown |
| *C12C8.2* | putative cystathionine gamma-lyase orthologous to human CTH |
| *C16A3.10* | ornithine aminotransferse precursor |
| *C17C3.1* | unknown |
| *C18A11.1* | unknown |
| *C32F10.4* | unknown |
| *C32H11.4* | unknown |
| *C34F11.8* | unknown |
| *C35A5.3* | sodium/phosphate transport protein |
| *C35A5.6* | unknown |
| *C35C5.9* | unknown |
| *C37H5.2* | unknown |
| *C39E9.8* | unknown |
| *C50F7.5* | unknown |
| *C55A6.6* | alcohol dehydrogenase |
| *cdr-4* | glutathione S-transferase |
| *cdr-7* | glutathione S-transferase |
| *cgp-1* | GTP-binding protein |
| *clec-1* | clec family, C-type lectin |
| *clec-67* | clec family, C-type lectin |
| *col-103* | collagen |
| *col-167* | collagen |
| *col-76* | collagen |
| *col-98* | collagen |
| *cpr-1* | cathepsin-like cysteine protease |
| *crn-2* | cell death related nuclease |
| *ctl-1* | catalase |
| *cyp-14A5* | cytochrome P450 |
| *cyp-25A1* | cytochrome P450 |
| *cyp-33C8* | cytochrome P450 |
| *dod-17* | unknown |
| *EGAP9.3* | unknown |
| *F09F7.6* | unknown |
| *F13A7.11* | unknown |
| *F13D12.3* | unknown |
| *F14F9.4* | unknown |
| *F15E6.4* | unknown |
| *F17A9.4* | unknown |
| *F21A3.2* | unknown |
| *F25E5.5* | F-box protein |
| *F32D8.12* | unknown |
| *F34H10.3* | protein S10 |
| *F35B12.3* | unknown |
| *F35E12.4* | unknown |
| *F36A2.3* | unknown |
| *F36F2.1* | unknown |
| *F43G6.8* | Zinc finger, C3HC4 type (RING finger) |
| *F45D3.4* | unknown |
| *F46F2.3* | unknown |
| *F52H3.5* | unknown |
| *F53A9.2* | unknown |
| *F54B8.4* | homolog of Death Associated Protein 1 (DAP-1) |
| *F55G11.4* | unknown |
| *F56D2.5* | unknown |
| *F56D5.3* | NADH oxidase |
| *F57C9.1* | unknown |
| *F59A7.2* | unknown |
| *F59F5.8* | unknown |
| *fbxa-128* | unknown |
| *fbxa-72* | unknown |
| *fbxa-79* | unknown |
| *fmo-3* | flavin-containing monoxygenase |
| *gcs-1* | gamma-glutamine cysteine synthetase heavy chain |
| *gpa-17* | unknown |
| *grd-5* | hedgehog like protein |
| *gst-1* | glutathione S-transferase |
| *gst-10* | glutathione S-transferase |
| *gst-12* | glutathione S-transferase |
| *gst-13* | glutathione S-transferase |
| *gst-14* | glutathione S-transferase |
| *gst-30* | glutathione S-transferase |
| *gst-38* | glutathione S-transferase |
| *gst-39* | glutathione S-transferase |
| *gst-4* | glutathione S-transferase |
| *hoe-1* | metal-dependent hydrolase orthologous to human ELAC2 |
| *K02A11.3* | placental protein 11 |
| *K11D12.5* | unknown |
| *K11H12.8* | unknown |
| *lec-11* | C type lectin |
| *lec-7* | C type lectin |
| *M153.1* | pyrroline-5-carboxylate reductase |
| *M60.4* | unknown |
| *math-36* | MATH (Meprin associated Traf homology) domain containing gene |
| *mbf-1* | Helix-turn-helix protein |
| *mtl-1* | metallothionein |
| *nhr-115* | zinc finger protein |
| *nhr-133* | zinc finger protein |
| *nhr-61* | zinc finger protein |
| *nhx-2* | NA(+)/H(+) exchanger |
| *pme-4* | glycohydrolase |
| *pqn-31* | glutamine/asparagine (Q/N)-rich ('prion') domain |
| *pqn-44* | glutamine/asparagine (Q/N)-rich ('prion') domain |
| *qdpr-1* | Alcohol/other dehydrogenases, short chain type |
| *R03E1.2* | unknown |
| *R03G5.5* | glutathione peroxidase |
| *R05D8.7* | unknown |
| *R06C7.5* | adenylosuccinate lyase |
| *R11F4.1* | Glycerol kinase |
| *R186.1* | unknown |
| *rpt-3* | triple A ATPase subunit of the 26S proteasome's 19S regulatory particle (RP) base subcomplex |
| *sdz-8* | alcohol dehydrogenase |
| *snb-1* | synaptobrevin |
| *sod-4* | Superoxide dismutase |
| *srr-4* | unknown |
| *T05C3.6* | unknown |
| *T05E7.4* | unknown |
| *T07D10.3* | zinc finger protein |
| *T12G3.1* | unknown |
| *T24C4.4* | unknown |
| *T28D6.3* | unknown |
| *T28F3.4* | Sugar (and other) transporters |
| *tag-234* | unknown |
| *tbb-6* | tubulin beta-chain |
| *tir-1* | TIR domain-containing protein SARM |
| *ugt-13* | ugt family |
| *ugt-19* | ugt family |
| *ugt-48* | ugt family |
| *ugt-62* | UDP-glucuronosyltransferase |
| *W05H9.1* | unknown |
| *W06H8.2* | unknown |
| *ZK228.4* | unknown |
