# Supplementary material for: Genomic Analysis of Stress Response against Arsenic in Caenorhabditis elegans
Source: PLoS One. 2013 Jul 24;8(7):e66431. doi: 10.1371/journal.pone.0066431 (PMC3722197; doi:10.1371/journal.pone.0066431)
Supplement: Table S6 — List of genes differentially expressed in both, high dose arsenic exposure and Elbe River sediment exposures (+/−1.5 fold). (DOCX) [file pone.0066431.s010.docx]

Table S6: List of genes differentially expressed in both, high dose arsenic exposure and Elbe River sediment exposures (+/- 1.5 fold).

| ***Gene Name*** | **Brief Description** |
| --- | --- |
| *abu-8* | glutamine/asparagine-rich domain and multiple cysteine-rich repeats (DUF139) |
| *alh-10* | aldehyde dehydrogenase |
| *ama-1* | RNA polymerase II |
| *amt-1* | ammonium transporter |
| *arf-1.2* | ADP-ribosylation factor homolog |
| *ark-1* | Src homology domain 3, tyrosine-protein kinase (ACK subfamily) |
| *atp-5* | ATP synthase D chain |
| *B0035.15* | unknown |
| *B0513.4* | unknown |
| *bli-4* | endoprotease |
| *C01G8.1* | unknown |
| *C02C6.3* | Leucine Rich Repeat (2 copies) |
| *C03H5.5* | unknown |
| *C04E12.4* | unknown |
| *C04F12.7* | unknown |
| *C10G8.3* | unknown |
| *C11G10.1* | unknown |
| *C16C8.2* | peroxidase precursor |
| *C17F4.3* | unknown |
| *C17H12.6* | unknown |
| *C18A11.1* | unknown |
| *C18B2.5* | unknown |
| *C18D11.1* | unknown |
| *C18E9.9* | unknown |
| *C18G1.9* | unknown |
| *C23G10.11* | unknown |
| *C24B9.3* | unknown |
| *C26D10.6* | unknown |
| *C27C12.4* | unknown |
| *C32F10.4* | unknown |
| *C33F10.12* | mitochondrial phosphate carrier protein |
| *C44B12.1* | unknown |
| *C44B7.10* | unknown |
| *C46C11.3* | unknown |
| *C47E12.3* | alpha-mannosidase |
| *C49F8.3* | unknown |
| *C50E3.6* | unknown |
| *calu-1* | calcium binding protein |
| *clec-47* | C-type lectin |
| *clec-5* | C-type lectin |
| *clec-72* | C-type lectin |
| *clec-85* | C-type lectin |
| *clec-87* | C-type lectin |
| *cls-2* | unknown |
| *cnc-4* | unknown |
| *col-19* | collagen |
| *col-38* | collagen |
| *cpi-1* | protease inhibitor |
| *cpt-1* | unknown |
| *crh-1* | bZIP transcription facto |
| *D1054.3* | unknown |
| *dhs-22* | dehydrogenase |
| *dlc-1* | dynein light chain 1 |
| *dlg-1* | guanylate kinase |
| *dod-3* | unknown |
| *dpy-2* | collagen |
| *F02C12.2* | glucose 1-dehydrogenase |
| *F09E5.7* | unknown |
| *F09F7.6* | unknown |
| *F10G7.5* | unknown |
| *F17H10.2* | unknown |
| *F18A11.3* | unknown |
| *F19B2.5* | unknown |
| *F22B7.9* | predicted methyltransferase |
| *F25A2.1* | unknown |
| *F26E4.6* | cytochrome C oxidase |
| *F26F12.3* | unknown |
| *F26H9.2* | unknown |
| *F28B3.10* | unknown |
| *F28E10.2* | unknown |
| *F28G4.4* | unknown |
| *F29G6.3* | unknown |
| *F30B5.4* | unknown |
| *F32B5.1* | phosphotransferase |
| *F36A2.7* | unknown |
| *F37A4.3* | unknown |
| *F38B6.4* | GARS/AIRS/GART |
| *F40F8.1* | UMP-CMP kinase like |
| *F40F9.5* | translocase |
| *F40G9.2* | unknown |
| *F41D3.11* | unknown |
| *F41G3.5* | protein kinase |
| *F44E2.6* | Transcription regulation |
| *F44G4.2* | unknown |
| *F45D3.2* | unknown |
| *F47B7.1* | unknown |
| *F48F5.1* | unknown |
| *F53A9.1* | unknown |
| *F53A9.8* | unknown |
| *F53F4.10* | NADH-ubiquinone dehydrogenase 24 KD subunit |
| *F57A8.1* | ETS domain |
| *F57B10.5* | unknown |
| *F58B3.6* | interferon-related protein PC4 like |
| *F59A1.11* | unknown |
| *F59F5.3* | tyrosine-protein kinase |
| *F59F5.8* | unknown |
| *fbxa-80* | unknown |
| *fbxa-98* | F-box containing protein |
| *fmo-1* | flavin-containing monoxygenase |
| *frm-7* | unknown |
| *ftn-2* | ferritin |
| *gei-7* | isocitrate lyase |
| *gly-8* | Glycosyl transferases |
| *grd-12* | hedgehog-like protein |
| *gst-7* | glutathione S-transferase |
| *H02I12.5* | unknown |
| *H10E21.1* | unknown |
| *hil-5* | histone H1 |
| *hot-5* | glycosylphosphatidylinositol (GPI)-linked signaling protein |
| *K03A1.4* | calmodulin calcium-binding sites |
| *K04G7.11* | unknown |
| *K07A12.7* | unknown |
| *K07B1.4* | unknown |
| *K08C7.6* | unknown |
| *K09F6.3* | protein-tyrosine phosphatase |
| *K10C3.2* | cAMP-regulated phosphoprotein |
| *K10H10.2* | beta-synthase |
| *kin-25* | protein tyrosine kinase |
| *klc-2* | kinesin light chain |
| *kpc-1* | Furin like serine protease |
| *lev-8* | acetylcholine receptor |
| *lgg-2* | Atg8/LC3 homolog |
| *M02B1.2* | unknown |
| *M88.2* | S34 subunit of the mitochondrial 28S ribosome |
| *math-26* | meprin-associated Traf homology (MATH) domain |
| *mbf-1* | Helix-turn-helix domain protein |
| *mel-32* | serine hydroxymethyltransferase |
| *msp-49* | major sperm protein |
| *nhr-21* | nuclear hormone receptor |
| *nhr-256* | zinc finger protein |
| *nhr-84* | zinc finger protein |
| *nhx-2* | NA(+)/H(+) exchanger |
| *nlp-29* | antimicrobial, neuropeptide-like protein |
| *npp-18* | WD domain, G-beta repeat (2 domains) |
| *nspd-4* | unknown |
| *pcn-1* | cyclin |
| *pcp-4* | peptidase |
| *pes-22* | predicted monocarboxylate porter subfamily of the major facilitator superfamily of transmembrane transporters |
| *pmt-2* | N-methyltransferase |
| *pqm-1* | Zinc finger, C2H2 type |
| *pqn-31* | glutamine/asparagine (Q/N)-rich ('prion') domain |
| *R08E5.1* | unknown |
| *R10D12.12* | unknown |
| *R11A5.3* | unknown |
| *R11D1.3* | unknown |
| *R12E2.6* | unknown |
| *R186.1* | unknown |
| *rig-6* | glutamine/asparagine (Q/N)-rich ('prion') domain |
| *rnh-1.3* | unknown |
| *rsp-5* | Alternative splicing factor SRp55/B52/SRp75 |
| *scp-1* | PTC-related protein that contains a sterol-sensing domain related to human Sterol regulatory element binding protein (SREBP) cleavage activating protein |
| *sft-1* | homolog of the human gene SURF1 |
| *snb-1* | synaptobrevin |
| *sodh-2* | alcohol dehydrogenase |
| *spn-4* | RNA-binding protein |
| *spp-1* | caenopore, a saposin (B) domain-containing protein |
| *sym-2* | hnRNP F protein like |
| *T01C3.3* | Zinc finger, C3HC4 type (RING finger) |
| *T01G9.2* | unknown |
| *T02E1.4* | unknown |
| *T05H4.4* | NADH-cytochrome B5 reductase |
| *T05H4.5* | NADH-cytochrome B5 reductase |
| *T07D10.3* | zinc finger protein |
| *T09B4.4* | unknown |
| *T12G3.1* | unknown |
| *T13H5.1* | protein-tyrosine phosphatase |
| *T16H12.9* | unknown |
| *T21H3.5* | unknown |
| *T22E7.1* | unknown |
| *T24B8.5* | ShK-like toxin peptide |
| *T24F1.3* | DAG binding domain |
| *T28B8.1* | unknown |
| *T28D6.3* | unknown |
| *T28F4.5* | homolog of Death Associated Protein 1 (DAP-1) protein |
| *tat-4* | ATPase |
| *tre-3* | trehalase |
| *ubxn-3* | UBX (ubiquitin regulatory X) domain-containing protein |
| *vha-1* | ATP synthase subunit C (2 domains) |
| *W01A11.1* | epoxide hydrolase |
| *W02D9.10* | unknown |
| *Y116F11B.13* | unknown |
| *Y17G7B.8* | unknown |
| *Y34B4A.5* | unknown |
| *Y39A1A.22* | unknown |
| *Y46D2A.2* | unknown |
| *Y51F10.2* | unknown |
| *Y51H1A.3* | NDUFB8, an accessory subunit of the mitochondrial respiratory chain NADH:ubiquinone oxidoreductase (Complex I); |
| *Y52B11A.3* | Heme-binding domain in cytochrome b5 and oxidoreductases |
| *Y57G11A.4* | unknown |
| *Y69A2AR.18* | unknown |
| *Y71F9B.13* | unknown |
| *ZC374.2* | Fibronectin type III domain (4 domains |
| *ZK1128.3* | unknown |
| *ZK185.2* | unknown |
