# Supplementary material for: Genomic Analysis of Stress Response against Arsenic in Caenorhabditis elegans
Source: PLoS One. 2013 Jul 24;8(7):e66431. doi: 10.1371/journal.pone.0066431 (PMC3722197; doi:10.1371/journal.pone.0066431)
Supplement: Table S7 — List of genes differentially expressed in both, high dose arsenic exposure and Rhine River sediment exposures (+/−1.5 fold). (DOCX) [file pone.0066431.s011.docx]

Table S7: List of genes differentially expressed in both, high dose arsenic exposure and Rhine River sediment exposures (+/- 1.5 fold).

| ***Gene Name*** | **Brief Description** |
| --- | --- |
| *abts-1* | anion exchange protein |
| *acl-14* | acyltransferase like |
| *acl-6* | glycerol-3-phosphate acyltransferase |
| *amt-1* | ammonium transporter |
| *B0222.3* | phosphate permease |
| *bec-1* | homolog of autophagy proteins Atg6/Vps30/Beclin1 |
| *C01B10.3* | paralog of IPP-5 |
| *C01F1.5* | unknown |
| *C02E7.6* | unknown |
| *C16H3.3* | unknown |
| *C18A11.1* | unknown |
| *C18B12.4* | Zinc finger, C3HC4 type (RING finger) |
| *C24A3.4* | E. coli L-carnitine dehydratase |
| *C26D10.6* | unknown |
| *C28C12.4* | unknown |
| *C32H11.1* | CUB (complement C1r/C1s, Uegf, Bmp1)-like domain protein |
| *C33F10.12* | mitochondrial phosphate carrier protein |
| *C34G6.1* | unknown |
| *C35A11.2* | unknown |
| *C36E8.1* | unknown |
| *C37A2.8* | unknown |
| *C42D4.13* | unknown |
| *C42D8.1* | unknown |
| *C43H8.1* | unknown |
| *C44B7.11* | unknown |
| *C44E4.1* | unknown |
| *calu-1* | calcium binding protein |
| *cdh-12* | cadherin |
| *cdh-7* | cadherin |
| *cex-2* | calcium binding protein |
| *cki-1* | homolog of the mammalian cyclin-dependent kinase inhibitor p27/KIP1 |
| *clec-47* | C-type lectin |
| *clec-86* | C-type lectin |
| *cpg-4* | unknown |
| *crn-2* | encodes a cell death-related nuclease, homologous to the magnesium-dependant TatD nuclease of E. coli |
| *cut-6* | cuticulin |
| *cyp-42A1* | cytochrome P450 |
| *D1044.1* | unknown |
| *D2085.7* | unknown |
| *dnj-2* | DNAJ protein like |
| *dpy-2* | collagen |
| *dyc-1* | homolog of murine CAPON, a protein associated with neuronal nitric oxide synthase |
| *ebp-2* | RP/EB-type microtubule-binding protein |
| *ech-2* | enoyl-CoA hydratase |
| *eps-8* | similarity to mouse epidermal growth factor receptor kinase substrate |
| *erm-1* | ERM family of cytoskeletal linker |
| *F01D4.3* | tyrosine-protein kinase (FES/FPS subfamily |
| *F08D12.2* | unknown |
| *F09A5.2* | tyrosine-protein kinase |
| *F10D7.2* | calcium alpha subunit; tetracycline resistance |
| *F10G7.10* | unknown |
| *F12B6.2* | sodium dependent-phosphate transporter |
| *F13D12.3* | unknown |
| *F13G3.11* | unknown |
| *F15D3.7* | unknown |
| *F15E6.3* | unknown |
| *F17H10.2* | unknown |
| *F21F8.11* | unknown |
| *F28E10.2* | unknown |
| *F28F5.4* | unknown |
| *F29G6.3* | unknown |
| *F31E3.2* | Protein kinase |
| *F37B4.10* | unknown |
| *F41D3.11* | unknown |
| *F41D3.7* | unknown |
| *F41G3.5* | protein kinase |
| *F43G6.3* | unknown |
| *F43G6.8* | Zinc finger, C3HC4 type (RING finger) |
| *F45E12.6* | unknown |
| *F46F5.6* | unknown |
| *F46G11.1* | orthologous to the human gene TRUNCATED PUTATIVE T7-LIKE MITOCHONDRIAL DNA HELICASE |
| *F47B8.3* | unknown |
| *F47G3.1* | unknown |
| *F48D6.4* | unknown |
| *F49H12.5* | unknown |
| *F52H2.4* | unknown |
| *F53C3.5* | unknown |
| *F53F1.3* | aldehyde reductase |
| *F56D1.1* | Zinc finger protein |
| *F57F4.4* | unknown |
| *F57F5.1* | cysteine protease |
| *F59F5.3* | tyrosine-protein kinase |
| *fbxa-51* | F-box motif containing protein |
| *flp-13* | unknown |
| *H24K24.3* | alcohol dehydrogenase |
| *hil-5* | histone H1 |
| *his-35* | histone H2A |
| *ifd-2* | cytoplasmic intermediate filament |
| *inx-8* | innexin family protein |
| *K02D3.2* | unknown |
| *K07A12.7* | unknown |
| *K07B1.4* | unknown |
| *K10B3.6* | unknown |
| *let-653* | mucin like protein |
| *M01H9.3* | unknown |
| *M02D8.2* | unknown |
| *M60.4* | unknown |
| *math-26* | meprin-associated Traf homology (MATH) domain |
| *mei-2* | unknown |
| *mel-32* | serine hydroxymethyltransferase |
| *mrp-5* | ABC transporter |
| *nas-11* | astacin-like metalloprotease |
| *nhr-136* | nuclear hormone receptor |
| *nhr-172* | nuclear hormone receptor |
| *nhr-183* | nuclear hormone receptor |
| *nhr-184* | nuclear hormone receptor |
| *nhr-50* | nuclear hormone receptor |
| *nhr-56* | nuclear hormone receptor |
| *nlp-13* | neuropeptide of the MSFamide family |
| *pcm-1* | L-isoaspartyl/D-aspartyl methyltransferase |
| *pcp-4* | peptidase |
| *R04F11.5* | unknown |
| *R08E5.1* | unknown |
| *R09D1.11* | chitinase |
| *R09H10.2* | unknown |
| *R10E9.2* | unknown |
| *R10H10.3* | CUB domain, von Willebrand factor type A domain |
| *R151.1* | unknown |
| *rme-6* | contains Ras-GAP (GTPase-activating protein)-like and Vps9 domains |
| *spp-16* | unknown |
| *srsx-28* | somatostatin receptor |
| *T02G5.11* | unknown |
| *T05H4.4* | NADH-cytochrome B5 reductase |
| *T05H4.5* | NADH-cytochrome B5 reductase |
| *T07D3.9* | unknown |
| *T09A5.7* | unknown |
| *T12B3.4* | unknown |
| *T12D8.9* | unknown |
| *T24D1.3* | unknown |
| *T25C12.3* | EGF-repeats |
| *T27A10.2* | unknown |
| *T27C4.2* | unknown |
| *T28F4.1* | unknown |
| *tag-52* | guanine-nucleotide releasing factor of the CDC24 family |
| *tre-2* | trehalase precursor |
| *ubxn-3* | UBX (ubiquitin regulatory X) domain-containing protein |
| *W09D10.3* | ribosomal protein L7/L12 mitochondrial |
| *W09D6.5* | unknown |
| *wnk-1* | serine/threonine kinase |
| *Y17G7B.8* | unknown |
| *Y32F6A.4* | unknown |
| *Y43B11AR.3* | unknown |
| *Y43C5A.3* | unknown |
| *Y43F8A.5* | unknown |
| *Y48G9A.9* | unknown |
| *Y51H1A.3* | ortholog of NDUFB8, an accessory subunit of the mitochondrial respiratory chain NADH:ubiquinone oxidoreductase (Complex I) |
| *Y58A7A.4* | unknown |
| *Y71F9B.2* | unknown |
| *Y71H10A.1* | phosphofructokinase |
| *ZK909.3* | guanosine-3',5'-bis(diphosphate)-pyrophosphohydrolase like |
| *ZK930.2* | unknown |
