# Supplementary material for: Genomic Analysis of Stress Response against Arsenic in Caenorhabditis elegans
Source: PLoS One. 2013 Jul 24;8(7):e66431. doi: 10.1371/journal.pone.0066431 (PMC3722197; doi:10.1371/journal.pone.0066431)
Supplement: Table S8 — Putative targets of top ten transcription factors that are predicted to be involving in the response to arsenic exposure by FastMEDUSA. (DOCX) [file pone.0066431.s012.docx]

Table S8: putative targets of top ten transcription factors that are predicted to be involving in the response to arsenic exposure by FastMEDUSA.

| **Transcription Factor** | | **putative target** |  | **Brief Description** |
| --- | --- | --- | --- | --- |
| B0412.1 | *(dac-1)* | B0222.4 | *(taq-38)* | glutamate decarboxylase |
|  |  | B0414.7 | *(mtk-1)* | protein kinase |
|  |  | C01G5.6 |  | homolog of Saccharomyces cerevisiae Ddi1/Vsm1 (DNA damage-inducible 1/v-SNARE-master 1) |
|  |  | C06C6.5 | *(nhr-50)* | Zinc finger, C4 type (two domains |
|  |  | C13C4.6 |  | unknown |
|  |  | C15H11.8 |  | DNA-directed RNA polymerase I like |
|  |  | C17C3.1 |  | unknown |
|  |  | C17H12.6 |  | unknown |
|  |  | C18H9.5 |  | sodium/phosphate transport protein |
|  |  | C30F12.6 | *(nmur-4)* | seven trans-membrane receptor |
|  |  | C33G3.3 | *(lgc-21)* | unknown |
|  |  | C42D4.1 |  | unknown |
|  |  | C42D4.3 |  | unknown |
|  |  | D2045.8 |  | TNF-alpha induced Protein B12 |
|  |  | F01G10.8 | *(daf-14)* | Mouse MSMAD protein like |
|  |  | F11G11.1 | *(gst-8)* | glutathione S-transferase |
|  |  | F20D1.6 | *(rbg-1)* | Human mRNA KIAA0066 predicted protein like |
|  |  | F28E10.2 |  | unknown |
|  |  | F33E2.2 | *(dlk-1)* | mitogen-activated protein kinase kinase kinase (MAPKKK) |
|  |  | F35E8.8 | *(gst-38)* | glutathione S-transferase |
|  |  | F36A2.3 |  | protein with similarity to malate dehydrogenase |
|  |  | F40G9.11 | *(mxl-2)* | unknown |
|  |  | F41E6.9 |  | unknown |
|  |  | F52E4.5 |  | unknown |
|  |  | F53A9.6 |  | unknown |
|  |  | F55G11.4 |  | unknown |
|  |  | F56H11.2 |  | unknown |
|  |  | H20E11.2 |  | unknown |
|  |  | K02A11.3 |  | placental protein 11 |
|  |  | K02F3.4 | *(zip-2)* | bZIP transcription factor |
|  |  | K08E7.7 | *(cul-6)* | culin family |
|  |  | K10C8.3 |  | unknown |
|  |  | R03G8.3 |  | unknown |
|  |  | R05F9.5 | *(gst-9)* | glutathione S-transferase |
|  |  | T06C12.6 | *(nhr-102)* | Zinc finger, C4 type (two domains) |
|  |  | T06D8.8 | *(rpn-9)* | non-ATPase subunit of the 19S regulatory complex of the proteasome |
|  |  | T06H11.4 | *(moc-1)* | gephyrin |
|  |  | T10H9.4 | *(snb-1)* | synaptobrevin |
|  |  | T19H5.4 |  | unknown |
|  |  | T22D1.9 | *(rpn-1)* | non-ATPase subunit of the 19S regulatory complex of the proteasome |
|  |  | T28B4.3 | *(ttr-6)* | unknown |
|  |  | T28B4.4 |  | claudin homolog |
|  |  | W02F12.4 |  | unknown |
|  |  | Y38C9A.2 | *(cgp-1)* | GTP-binding protein |
|  |  | Y38E10A.12 | *(nspe-3)* | unknown |
|  |  | Y38E10A.15 | *(nspe-7)* | unknown |
|  |  | Y42G9A.3 |  | unknown |
|  |  | Y4C6B.3 |  | unknown |
|  |  | Y52E8A.3 |  | unknown |
|  |  | Y69H2.3 |  | unknown |
|  |  | Y73B6BL.4 |  | unknown |
|  |  | Y97E10AR.6 |  | unknown |
|  |  | ZC13.1 |  | zinc finger protein |
|  |  | ZC376.7 | *(atfs-1)* | DNA binding domain |
|  |  | ZK822.5 |  | sodium/proline symporter like |
|  |  | ZK945.2 | *(pas-7)* | proteasome component (A-type |
| C07A12.3 | *(nhr-35)* | B0213.4 | *(nlp-29)* | antimicrobial, neuropeptide-like protein |
|  |  | B0414.8 | *(vps-51)* | unknown |
|  |  | C09B8.4 |  | unknown |
|  |  | C12D8.11 | *(rop-1)* | ribonucleoprotein RO autoantigen |
|  |  | C13C4.6 |  | unknown |
|  |  | C13D9.1 | *(srr-6)* | unknown |
|  |  | C14A4.1 | *(dohh-1)* | unknown |
|  |  | C17H11.6 |  | SR-famC |
|  |  | C18B12.4 |  | Zinc finger, C3HC4 type (RING finger) |
|  |  | C23G10.4 | *(rpn-2)* | tRNA processing protein SEN3 |
|  |  | C30C11.2 | *(rpn-3)* | Diphenol oxidase A2 |
|  |  | C49G7.12 |  | unknown |
|  |  | D1014.3 | *(snap-1)* | alpha-SNAP protein |
|  |  | F01D5.1 |  | unknown |
|  |  | F02A9.1 |  | unknown |
|  |  | F15A8.6 |  | esterase |
|  |  | F19H8.1 | *(tps-2)* | trehalose phosphate synthase |
|  |  | F22B7.7 | *(twk-7)* | Potassium channel protein |
|  |  | F22B7.9 |  | predicted methyltransferase |
|  |  | F22D6.2 |  | unknown |
|  |  | F25B5.4 | *(ubq-1)* | unknown |
|  |  | F32G8.6 | *(cat-4)* | GTP cyclohydrolase I |
|  |  | F35C11.6 |  | unknown |
|  |  | F35E8.8 | *(gst-38)* | glutathione S-transferase |
|  |  | F37B1.2 | *(gst-12)* | glutathione S-transferase |
|  |  | F38A6.1 | *(pha-4)* | Fork head domain, eukaryotic transcription factors |
|  |  | F40F8.7 | *(pqm-1)* | Zinc finger, C2H2 type |
|  |  | F41E7.1 |  | unknown |
|  |  | F54B8.3 | *(fbxa-69)* | F-box motif containing protein |
|  |  | F54B8.4 |  | homolog of Death Associated Protein 1 (DAP-1) |
|  |  | K01H12.1 | *(dph-3)* | unknown |
|  |  | R05F9.1 |  | yeast suppressor protein SRP40 |
|  |  | R107.7 | *(gst-1)* | glutathione S-transferase |
|  |  | R166.5 | *(mnk-1)* | serine/threonine kinase |
|  |  | T01E8.1 |  | unknown |
|  |  | T10D4.3 |  | unknown |
|  |  | T10G3.1 |  | unknown |
|  |  | T10H9.4 | *(snb-1)* | synaptobrevin |
|  |  | T20F10.1 | *(wts-1)* | Protein kinase C terminal domain |
|  |  | T28B4.3 | *(ttr-6)* | unknown |
|  |  | W06H8.2 |  | unknown |
|  |  | Y37E11AR.4 | *(nape-1)* | unknown |
|  |  | Y42G9A.3 |  | unknown |
|  |  | Y43F8B.2 |  | unknown |
|  |  | Y4C6B.3 |  | unknown |
|  |  | Y70C5C.2 | *(clec-9)* | C-type lectin |
|  |  | ZC376.7 | *(atfs-1)* | DNA binding domain |
|  |  | ZK546.11 | *(gst-30)* | glutathione S-transferase |
| C32D5.1 |  | C06G3.6 |  | unknown |
|  |  | C07G3.2 | *(irg-1)* | protein containing a conserved DUF1768 domain |
|  |  | C17C3.1 |  | unknown |
|  |  | C18H9.5 |  | sodium/phosphate transport protein |
|  |  | C28C12.4 |  | unknown |
|  |  | C30C11.4 |  | member of the Hsp70 family of heat shock proteins |
|  |  | C30F12.6 | *(nmur-4)* | seven trans-membrane receptor |
|  |  | C33G3.3 | *(lgc-21)* | unknown |
|  |  | C50F4.7 | *(his-37)* | histone H4 |
|  |  | C55A6.6 |  | alcohol dehydrogenase |
|  |  | F08F3.7 | *(cyp-14A5)* | cytochrome P450 |
|  |  | F09C8.2 |  | unknown |
|  |  | F10D7.5 |  | ortholog of Drosophila NEURALIZED |
|  |  | F26G1.2 |  | unknown |
|  |  | F28E10.2 |  | unknown |
|  |  | F28H1.1 |  | unknown |
|  |  | F37B1.2 | *(gst-12)* | glutathione S-transferase |
|  |  | F41C3.2 |  | Sodium/phosphate transporter protein |
|  |  | F55G1.9 |  | carboxylate reductase |
|  |  | F56H1.4 | *(rpt-5)* | ATPase |
|  |  | H19N07.3 |  | unknown |
|  |  | H32C10.2 | *(lin-33)* | unknown |
|  |  | R02E4.1 |  | unknown |
|  |  | R05F9.5 | *(gst-9)* | glutathione S-transferase |
|  |  | T01G6.6 | *(nhr-212)* | nuclear hormone receptor |
|  |  | T06D8.8 | *(rpn-9)* | non-ATPase subunit of the 19S regulatory complex of the proteasome |
|  |  | T10F2.2 |  | orthologous to the human gene SOLUTE CARRIER FAMILY 25 (MITOCHONDRIAL CARRIER; ORNITHINE TRANSPORTER) |
|  |  | T22D1.9 | *(rpn-1)* | non-ATPase subunit of the 26S proteasome's 19S regulatory particle (RP) base subcomplex |
|  |  | T27A3.2 |  | ubiquitin carboxyl-terminal hydrolase |
|  |  | T28F3.4 |  | Sugar (and other) transporters |
|  |  | T28H10.1 |  | B.subtilis SFP protein like |
|  |  | Y116F11B.12 | *(gly-4)* | UDP-GalNAc:polypeptide N-acetylgalactosaminyltransferase |
|  |  | Y42G9A.3 |  | unknown |
|  |  | Y77E11A.2 |  | unknown |
|  |  | ZK20.1 | *(ghi-1)* | member of a family of lipid and lipopolysaccharide-binding proteins |
|  |  | ZK525.2 | *(aqp-11)* | putative aquaporin |
|  |  | ZK945.2 | *(pas-7)* | proteasome component (A-type) |
| F16H11.5 | *(nhr-45)* | B0205.11 | *(mrpl-9)* | unknown |
|  |  | C01B12.3 |  | orthologous to the human gene VITELLIFORM MACULAR DYSTROPHY PROTEIN |
|  |  | C06G3.1 | *(nhr-105)* | zinc finger protein |
|  |  | C18B12.4 |  | Zinc finger, C3HC4 type (RING finger) |
|  |  | C30C11.4 |  | member of the Hsp70 family |
|  |  | C30F12.6 | *(nmur-4)* | seven trans-membrane receptor |
|  |  | C41H7.7 | *(clec-3)* | unknown |
|  |  | C42D4.3 |  | unknown |
|  |  | C50F4.7 | *(his-37)* | histone H4 |
|  |  | C54D10.1 | *(cdr-2)* | glutathione S-transferase |
|  |  | F01G10.8 | *(daf-14)* | Mouse MSMAD protein like |
|  |  | F09C6.1 |  | unknown |
|  |  | F09F3.6 | *(ttr-21)* | Transthyretin-like family |
|  |  | F10D7.5 |  | ortholog of Drosophila NEURALIZED |
|  |  | F11G11.2 | *(gst-7)* | glutathione S-transferase |
|  |  | F22B7.7 | *(twk-7)* | Potassium channel protein |
|  |  | F26E4.12 |  | glutathione peroxidase |
|  |  | F26F12.3 |  | unknown |
|  |  | F31F4.12 | *(nhr-18)* | zinc finger protein |
|  |  | F31F7.1 |  | unknown |
|  |  | F32A6.4 | *(ags-3)* | Activator of G Protein Signaling 3 (AGS3) family |
|  |  | F36F2.1 |  | unknown |
|  |  | F37B1.2 | *(gst-12)* | glutathione S-transferase |
|  |  | F37C12.10 |  | unknown |
|  |  | F41B5.9 | *(nhr-182)* | nuclear hormone receptor |
|  |  | F44F4.4 | *(ptr-8)* | sterol sensing domain (SSD) protein |
|  |  | F47B8.2 |  | unknown |
|  |  | F53B2.8 |  | unknown |
|  |  | F54B8.4 |  | homolog of Death Associated Protein 1 (DAP-1) |
|  |  | F58B4.1 | *(nas-31)* | zinc metalloprotease |
|  |  | K08F4.11 | *(gst-3)* | glutathione S-transferase |
|  |  | K11G9.5 |  | unknown |
|  |  | M02F4.7 | *(clec-265)* | C-type lectin |
|  |  | R03G8.3 |  | unknown |
|  |  | T04C10.4 | *(atf-5)* | transcription factor ATF4 lik |
|  |  | T20B5.3 | *(oga-1)* | ortholog of mammalian O-linked N-acetylglucosamine (O-GlcNAc)-selective N-acetyl-beta-D-glucosaminidase (O-GlcNAcase) |
|  |  | T27A3.2 |  | ubiquitin carboxyl-terminal hydrolase |
|  |  | T28B11.1 |  | F-box motif containing protein |
|  |  | T28B4.3 | *(ttr-6)* | unknown |
|  |  | T28H11.8 |  | phosphate cotransporter |
|  |  | W01D2.2 | *(nhr-61)* | Zinc finger, C4 type (two domains |
|  |  | W10G11.1 |  | unknown |
|  |  | Y22F5A.1 | *(nhr-232)* | Zinc finger, C4 type (two domains) |
|  |  | Y34D9A.10 | *(vps-4)* | unknown |
|  |  | Y37H9A.1 |  | unknown |
|  |  | Y38E10A.12 | *(nspe-3)* | unknown |
|  |  | Y38E10A.13 | *(nspe-1)* | unknown |
|  |  | Y48E1B.10 | *(gst-20)* | glutathione S-transferase |
|  |  | Y4C6B.3 |  | unknown |
|  |  | Y57A10A.14 |  | unknown |
|  |  | Y71F9AL.10 |  | unknown |
|  |  | Y75B12A.2 |  | unknown |
|  |  | ZC13.1 |  | zinc finger protein |
|  |  | ZK228.4 |  | unknown |
|  |  | ZK673.9 | *(clec-143)* | C-type lectin |
|  |  | ZK75.1 | *(ins-4)* | insulin-like peptide |
| F38A5.13 | *(dnj-11)* | B0554.5 |  | unknown |
|  |  | C06G3.1 | *(nhr-105)* | zinc finger protein |
|  |  | C13C4.6 |  | unknown |
|  |  | C14F11.3 | *(lite-1)* | eight-transmembrane protein |
|  |  | C17H11.6 |  | SR-famC |
|  |  | C18B12.4 |  | Zinc finger, C3HC4 type (RING finger) |
|  |  | C42D4.3 |  | unknown |
|  |  | C49G7.12 |  | unknown |
|  |  | D1014.3 | *(snap-1)* | alpha-SNAP protein |
|  |  | E01G6.1 |  | pancreatic trypsin inhibitor (kunitz) protein |
|  |  | F25B5.4 | *(ubq-1)* | unknown |
|  |  | K01H12.1 | *(dph-3)* | unknown |
|  |  | M03A8.2 | *(atg-2)* | homolog of yeast protein Atg2p |
|  |  | M176.2 | *(gss-1)* | glutathione synthase |
|  |  | R166.5 | *(mnk-1)* | serine/threonine kinase |
|  |  | T10H9.4 | *(snb-1)* | synaptobrevin |
|  |  | Y38E10A.12 | *(nspe-3)* | unknown |
|  |  | Y57G11C.4 | *(vti-1)* | unknown |
| F40G9.11 | *(mxl-2)* | C05B5.8 |  | unknown |
|  |  | C08F11.13 |  | unknown |
|  |  | C14F11.3 | *(lite-1)* | eight-transmembrane protein that is a member of the invertebrate family of Gustatory receptors |
|  |  | C18B2.4 |  | unknown |
|  |  | C29F3.3 |  | unknown |
|  |  | C30C11.4 |  | Hsp70 family of heat shock proteins |
|  |  | C37H5.8 | *(hsp-6)* | heat shock 70 protein |
|  |  | C46F4.2 | *(acs-17)* | long-chain-fatty-acid coA ligase |
|  |  | C55F2.2 | *(ilys-4)* | unknown |
|  |  | F01D5.5 |  | unknown |
|  |  | F09F3.6 | *(ttr-21)* | Transthyretin-like family |
|  |  | F20D1.10 | *(tag-299)* | unknown |
|  |  | F22B7.7 | *(twk-7)* | Potassium channel protein |
|  |  | F35E8.8 | *(gst-38)* | glutathione S-transferase |
|  |  | F52E4.5 |  | unknown |
|  |  | F55A12.7 | *(apm-1)* | clathrin coat assembly protein complex 1 medium chain |
|  |  | F56C3.9 |  | unknown |
|  |  | F56C9.10 |  | unknown |
|  |  | K04G7.3 | *(ogt-1)* | ortholog of O-linked N-acetylglucosamine (O-GlcNAc) transferase |
|  |  | K08D8.3 |  | unknown |
|  |  | M03A8.2 | *(atg-2)* | orthologous to the autophagic budding yeast protein Atg2p |
|  |  | M60.6 |  | unknown |
|  |  | R02E4.1 |  | unknown |
|  |  | R09B5.9 | *(cnc-4)* | caenacin peptide |
|  |  | T04C10.4 | *(atf-5)* | homolog of the mammalian bZIP transcription factors ATF4 and ATF5 |
|  |  | T05E7.4 |  | unknown |
|  |  | T07C4.9 | *(nex-2)* | Annexin |
|  |  | T10F2.2 |  | Mitochondrial carrier protein |
|  |  | W05H9.3 |  | unknown |
|  |  | W06H8.2 |  | unknown |
|  |  | Y26E6A.1 | *(ekl-5)* | unknown |
|  |  | Y38E10A.12 | *(nspe-3)* | unknown |
|  |  | ZK525.2 | *(aqp-11)* | putative aquaporin |
|  |  | ZK546.11 | *(gst-30)* | lutathione S-transferase |
|  |  | ZK945.2 | *(pas-7)* | proteasome component (A-type |
| R06C1.6 |  | C02C6.2 | *(olrn-1)* | orthologous to Drosophila melanogaster RAW and Schistosoma japonicum SJCHGC05616 |
|  |  | C03A7.11 | *(ugt-51)* | 7TM chemoreceptor, ugt family |
|  |  | C04F12.1 |  | unknown |
|  |  | C07G3.2 | *(irg-1)* | protein containing a conserved DUF1768 domain |
|  |  | C50F4.7 | *(his-37)* | histone H4 |
|  |  | C54F6.4 | *(swt-2)* | unknown |
|  |  | D2013.9 | *(ttll-12)* | putative tubulin polyaminoacid ligase (possibly a polyglycylase) orthologous to human TTLL12 |
|  |  | F37B1.5 | *(gst-16)* | glutathione S-transferase |
|  |  | F53A9.8 |  | unknown |
|  |  | F55G1.9 |  | carboxylate reductase |
|  |  | F56C9.10 |  | unknown |
|  |  | T01G6.6 | *(nhr-212)* | nuclear hormone receptor |
|  |  | T05A7.1 |  | unknown |
|  |  | T21F4.1 |  | orthologous to the human gene ARGINASE TYPE I ERYTHROID VARIANT |
|  |  | T26C5.1 | *(gst-13)* | glutathione S-transferase |
|  |  | T28F4.1 |  | unknown |
|  |  | Y46H3A.3 | *(hsp-16.2)* | heat shock protein |
|  |  | ZK632.6 | *(cnx-1)* | Calnexin |
|  |  | ZK945.2 | *(pas-7)* | proteasome component (A-type) |
| W01D2.2 | *(nhr-6)* | B0511.6 |  | helicase |
|  |  | C04F12.1 |  | unknown |
|  |  | C08F11.13 |  | unknown |
|  |  | C14A4.1 | *(dohh-1)* | unknown |
|  |  | C14F11.3 | *(lite-1)* | encodes a eight-transmembrane protein that is a member of the invertebrate family of Gustatory receptors |
|  |  | C16A11.2 |  | unknown |
|  |  | C17H11.6 |  | SR-famC |
|  |  | C18B2.4 |  | unknown |
|  |  | C27B7.4 | *(rad-26)* | DNA repair protein |
|  |  | C27C12.2 | *(egrh-1)* | Zinc finger, C2H2 type (3 domains) |
|  |  | C28C12.10 | *(tag-77)* | unknown |
|  |  | C32F10.1 | *(obr-4)* | oxysterol-binding protein |
|  |  | C49G7.5 | *(irg-2)* | unknown |
|  |  | C50F4.7 | *(his-37)* | histone H4 |
|  |  | C55F2.2 | *(ilys-4)* | unknown |
|  |  | E01G6.1 |  | pancreatic trypsin inhibitor (kunitz) protein |
|  |  | F08G2.4 |  | unknown |
|  |  | F17C11.11 |  | unknown |
|  |  | F20D1.10 | *(tag-299)* | unknown |
|  |  | F22B7.9 |  | predicted methyltransferase |
|  |  | F23C8.6 |  | unknown |
|  |  | F29G9.4 | *(fos-1)* | BZIP transcription facto |
|  |  | F35B3.4 |  | unknown |
|  |  | F37C4.5 |  | unknown |
|  |  | F41B5.10 | *(nhr-183)* | nuclear hormone receptor |
|  |  | F41E6.9 |  | unknown |
|  |  | F46C3.1 | *(pek-1)* | eukaryotic translation initiation factor 2 alpha kinase PEK |
|  |  | F48F7.7 | *(arrd-24)* | unknown |
|  |  | F52E4.5 |  | unknown |
|  |  | F53A9.6 |  | unknown |
|  |  | F54E2.1 |  | unknown |
|  |  | K04F1.9 |  | unknown |
|  |  | K05C4.1 | *(pbs-5)* | Proteasome A-type and B-type |
|  |  | K05C4.2 |  | unknown |
|  |  | R10E12.1 | *(alx-1)* | Bro1 domain-containing protein |
|  |  | R166.3 |  | orthologous to the human gene ALPORT SYNDROME, MENTAL RETARDATION, MIDFACE HYPOPLASIA, AND ELLIPTOCYTOSIS CHROMOSOMAL REGION GENE 1 |
|  |  | T04C10.4 | *(atf-5)* | transcription factor ATF4 like |
|  |  | T14E8.1 |  | protein-tyrosine kinase |
|  |  | T25G3.3 |  | Yeast nonsense-mediated mRNA decay protein like |
|  |  | Y17G7B.13 |  | unknown |
|  |  | Y38E10A.15 | *(nspe-7)* | unknown |
|  |  | Y48G9A.9 |  | unknown |
|  |  | Y77E11A.2 |  | unknown |
|  |  | ZK546.11 | *(gst-30)* | glutathione S-transferase |
|  |  | ZK945.2 | *(pas-7)* | proteasome component (A-type |
| Y44E3B.1 | *(zip-4)* | C01G10.8 |  | unknown |
|  |  | C04F12.1 |  | unknown |
|  |  | C06B3.6 |  | unknown |
|  |  | C06G3.1 | *(nhr-105)* | zinc finger protein |
|  |  | C14F11.3 | *(lite-1)* | encodes a eight-transmembrane protein that is a member of the invertebrate family of Gustatory receptors |
|  |  | C17C3.1 |  | unknown |
|  |  | C28C12.10 | *(tag-77)* | unknown |
|  |  | C29F3.3 |  | unknown |
|  |  | C30C11.4 |  | Msi3p |
|  |  | C37H5.3 |  | unknown |
|  |  | D1014.3 | *(snap-1)* | alpha-SNAP protein |
|  |  | E01G6.1 |  | pancreatic trypsin inhibitor (kunitz) protein |
|  |  | F09C6.3 |  | unknown |
|  |  | F09C8.2 |  | unknown |
|  |  | F09F3.6 | *(ttr-21)* | Transthyretin-like family |
|  |  | F13B10.1 | *(tir-1)* | SAM domain (Sterile alpha motif |
|  |  | F14D12.5 | *(sulp-2)* | sulfate transporter |
|  |  | F20C5.6 |  | spectrin |
|  |  | F22B7.9 |  | encodes a predicted methyltransferase |
|  |  | F23C8.6 |  | unknown |
|  |  | F28H1.1 |  | unknown |
|  |  | F33E2.2 | *(dlk-1)* | encodes a mitogen-activated protein kinase kinase kinase (MAPKKK) |
|  |  | F35B3.4 |  | unknown |
|  |  | F36A2.3 |  | encodes a protein with similarity to malate dehydrogenase |
|  |  | F41B5.10 | *(nhr-183)* | nuclear hormone receptor |
|  |  | F41G4.8 |  | unknown |
|  |  | F46C3.1 | *(pek-1)* | eukaryotic translation initiation factor 2 alpha kinase PEK |
|  |  | F48F7.7 | *(arrd-24)* | unknown |
|  |  | F53C3.5 |  | unknown |
|  |  | F55G11.4 |  | unknown |
|  |  | R01E6.4 | *(acr-12)* | acetylcholine receptor protein |
|  |  | T10H9.4 | *(snb-1)* | synaptobrevin |
|  |  | T14E8.1 |  | protein-tyrosine kinase |
|  |  | T20F5.2 | *(pbs-4)* | peptidase |
|  |  | T25G3.3 |  | Yeast nonsense-mediated mRNA decay protein like |
|  |  | T26C5.1 | *(gst-13)* | glutathione S-transferase |
|  |  | T27A3.2 |  | ubiquitin carboxyl-terminal hydrolase |
|  |  | T28B4.3 | *(ttr-6)* | unknown |
|  |  | W01D2.2 | *(nhr-61)* | Zinc finger, C4 type (two domains) |
|  |  | Y116F11B.12 | *(gly-4)* | UDP-GalNAc:polypeptide N-acetylgalactosaminyltransferase |
|  |  | Y38E10A.15 | *(nspe-7)* | unknown |
|  |  | Y43B11AR.3 |  | unknown |
|  |  | Y46H3A.3 | *(hsp-16-2)* | heat shock protein |
|  |  | Y48G9A.9 |  | unknown |
|  |  | Y59H11AR.4 |  | unknown |
|  |  | ZC13.1 |  | zinc finger protein |
| Y48C3A.4 | *(ztf-22)* | B0554.5 |  | unknown |
|  |  | C10H11.4 | *(ugt-28)* | ugt family |
|  |  | C14A4.1 | *(dohh-1)* | unknown |
|  |  | C30C11.2 | *(rpn-3)* | Diphenol oxidase A2 |
|  |  | C50F4.7 | *(his-37)* | histone H4 |
|  |  | F08G12.5 |  | Zinc finger, C3HC4 type (RING finger |
|  |  | F22B7.9 |  | predicted methyltransferase |
|  |  | F23C8.6 |  | protein containing N-terminal TRP domains and C-terminal GPR domains that is a member of the Activator of G Protein Signaling 3 (AGS3) family |
|  |  | F32A6.4 | *(ags-3)* | protein containing N-terminal TRP domains and C-terminal GPR domains that is a member of the Activator of G Protein Signaling 3 (AGS3) family |
|  |  | F35E8.8 | *(gst-38)* | glutathione S-transferase |
|  |  | F37C12.10 |  | unknown |
|  |  | F47B8.3 |  | unknown |
|  |  | F55A12.7 | *(apm-1)* | clathrin coat assembly protein complex 1 medium chain |
|  |  | F56C3.9 |  | unknown |
|  |  | K07D4.3 | *(rpn-11)* | predicted non-ATPase subunit of the 19S regulatory complex of the proteasome |
|  |  | T01G6.6 | *(nhr-212)* | nuclear hormone recepto |
|  |  | Y32H12A.3 | *(dhs-9)* | dehydrogenase |
|  |  | Y38H6C.8 |  | Lectin C-type domain short and long forms |
|  |  | Y48A6B.7 |  | Cytidine and deoxycytidylate deaminases zinc-binding region |
|  |  | ZC376.7 | *(atfs-1)* | DNA binding domain |
|  |  | ZK945.2 | *(pas-7)* | proteasome component (A-type) |
